# Memristor-Based Artificial Neural Networks for Hardware Neuromorphic Computing

**DOI:** 10.34133/research.0758

**Published:** 2025-07-04

**Authors:** Boyan Jin, Zhenlong Wang, Tianyu Wang, Jialin Meng

**Affiliations:** ^1^ School of Integrated Circuits, Shandong University, Jinan 250101, China.; ^2^ Shenzhen Research Institute of Shandong University, Shenzhen 518100, China.; ^3^ National International Innovation Center, Shanghai 201203, China.; ^4^ State Key Laboratory of Crystal Materials, Shandong University, Jinan 250100, China.; ^5^ Key Laboratory of Computational Neuroscience and Brain-Inspired Intelligence (Fudan University), Ministry of Education, Shanghai 200433, China.

## Abstract

Artificial neural networks have long been studied to emulate the cognitive capabilities of the human brain for artificial intelligence (AI) computing. However, as computational demands intensify, conventional hardware based on transistor and complementary metal oxide semiconductor (CMOS) technology faces substantial limitations due to the separation of memory and processing, a challenge commonly known as the von Neumann bottleneck. In this review, we examine how memristors, which are novel nonvolatile memory devices that exhibit memory-dependent resistance, can be harnessed to build more efficient and scalable neural networks. We provide a comprehensive background on the evolution of neural network models and memristors, as well as introduce the principles of memristive devices, which mimic the dynamic behavior of biological synapses. Various neural network architectures, including convolutional, recurrent, and spiking models, are discussed, highlighting the advantages of integrating memristors for in-memory computing and parallel processing. Our review further examines key mechanisms such as synaptic plasticity, encompassing both long-term potentiation and depression, as well as emerging learning algorithms that leverage memristive behavior. Finally, we identify current challenges, such as achieving ultra-low power consumption, high device uniformity, and seamless system integration, and propose future directions in materials science, device engineering, system integration, and industrialization. These advances suggest that memristor-based neural networks may pave the way for next-generation AI systems that combine low power consumption with high computational performance, ultimately bridging the gap between biological and electronic information processing.

## Introduction

The study of artificial neural networks (ANNs) stems from the enthusiasm for drilling in the field of artificial intelligence (AI) since the 1940s, which has driven scientists to explore the mechanisms of how to simulate the human mind. From the mathematical modeling of neurons by McCulloch and Pitts in 1943 [1], to a creative gathering in 1956 at Dartmouth University, New Hampshire, USA, led by McCarthy, Minsky, Shannon, Newell, and Simon, under the name of Artificial Intelligence, the concept of “artificial intelligence” was coined. “Artificial intelligence” concept, and then the subsequent Rosenblatt perceptron [2], Hopfield network [3], and BP algorithm [4] and other technological innovations, the development of ANN has always been closely centered on the achievement of the ambitious goal of machine intelligence. It is under the extensive promotion of the interdisciplinary field of AI, neural networks, as the core method of simulating the brain to process information not only lays the theoretical foundation for computers to think like human beings but also promotes the development of a variety of applications ranging from chess games to associative memory, pattern recognition, etc., and has become an important pillar of the overall progress of AI technology.

After over 80 years of evolution, ANNs have flourished with numerous specialized branches. Established frameworks include the convolutional neural network (CNN) [5], which originated from the “Neocognitron” model proposed by Fukushima in 1979; the recurrent neural network (RNN) [6], developed in the 1990s through the integration of Elman networks by Elman and long short-term memory (LSTM) networks by Schmidhuber; the spiking neural network (SNN), evolved from Lapicque’s integrate-and-fire model (1907) [7] and the biologically inspired Hodgkin–Huxley spiking neuron model (1952) [8]; the generative adversarial network (GAN) [9], introduced by Ian Goodfellow and colleagues in 2014; and the Transformer model without recurrent units [10], presented by Vaswani et al. in 2017.

With technological advancement, the neuromorphic brain-inspired computing paradigm, which simulates neuronal and synaptic behaviors through transistors and complementary metal oxide semiconductor (CMOS) integrated circuits, has reached relative maturity. Despite various algorithmic and architectural innovations, the training efficiency of ANNs on large datasets faces hardware limitations due to the von Neumann bottleneck (data transfer latency outpacing computation speed) and the slowing pace of device miniaturization under Moore’s law. This technological impasse necessitates novel solutions for computational hardware and architecture. In this context, emerging devices and materials have gained prominence, particularly novel nonvolatile memories (NVMs) employing resistive switching mechanisms, such as resistive random-access memory (RRAM) and conductive-bridging random-access memory (CBRAM). These passive electronic components with memristive properties, termed memristors, were first theorized by Professor Chua as the fourth fundamental circuit element [11], later physically realized by HP Labs through a $\mathrm{Ti}O_{2}$-based RRAM device [12]. Memristors have emerged as pivotal materials for neural network hardware implementations due to their compact size and low energy consumption, effectively addressing escalating computational demands.

**Fig. 1.**
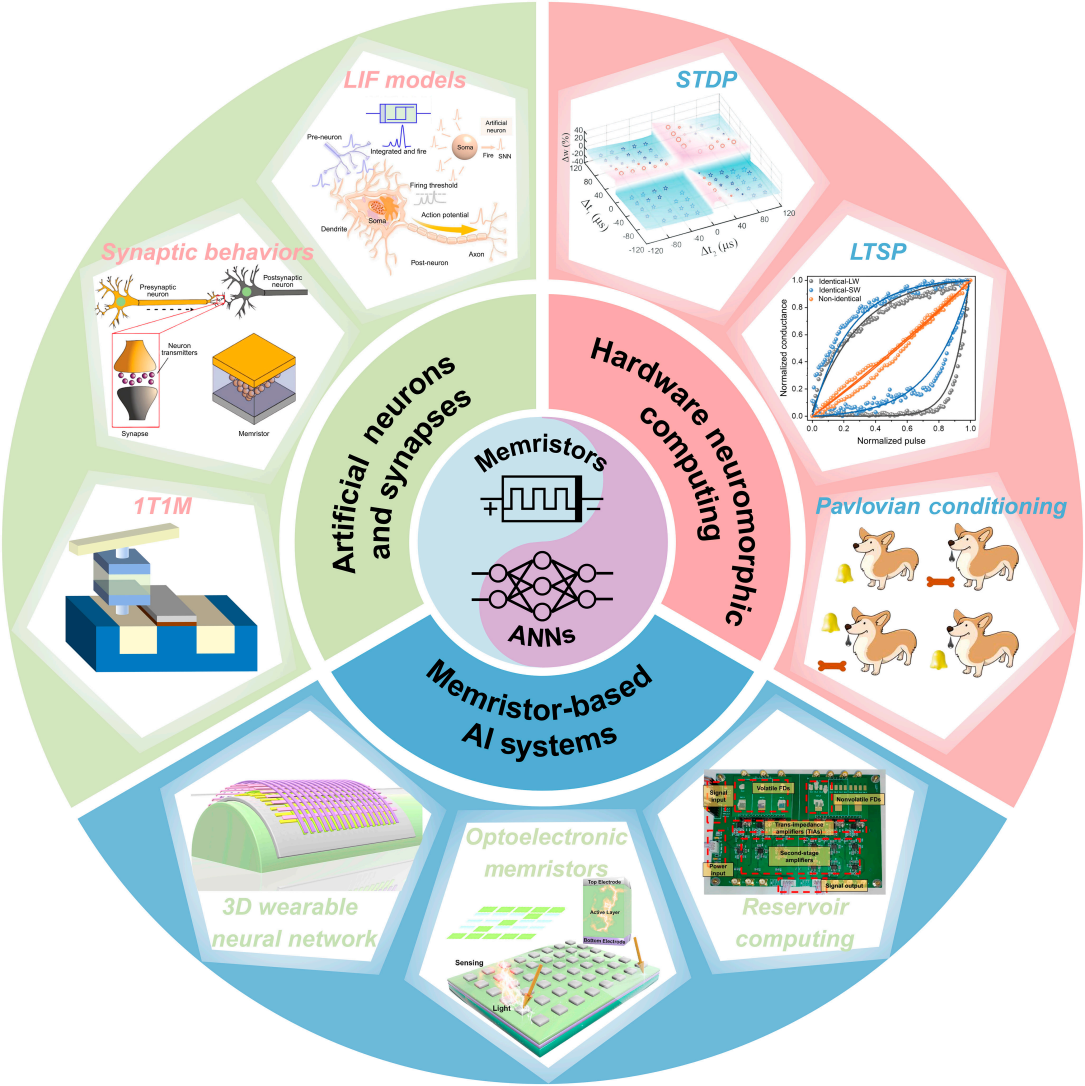
Overview of memristor-based neural network computing. Memristors and neural networks have evolved in close correlation; the center of the overview diagram displays the symbolic representation of memristors alongside a conceptual illustration of the input, hidden, and output layers of an ANN. In the “Artificial Neurons and Synapses” module, the LIF models are presented (reproduced under the terms of the CC BY 4.0 license [44]; copyright 2022, The Authors, published by Springer Nature), featuring a comparative schematic of biological and artificial neurons along with their pulse response results; the module also illustrates synaptic behaviors (reproduced under the terms of the CC BY 4.0 license [137]; copyright 2022, The Authors, published by Wiley), demonstrating how artificial synapses emulate biological synapses and the method of synaptic neurotransmitter transmission, and showcases the 1T1M structure, which involves a series connection of a transistor and a memristor with a MIM structure. In the “Hardware Neuromorphic Computing” module, a schematic of triplet spike timing-dependent (triplet-STDP) plasticity responses is presented (reproduced with permission from [138]; copyright 2017, Wiley), which details the dependency of second-order memristors on synaptic weights across 4 temporal quadrants; the module also exhibits a schematic of LTSP (reproduced under the terms of the CC BY 4.0 license [106]; copyright 2022, The Authors, published by Wiley), highlighting the variations of LTP and LTD under 3 different pulse conditions, and provides a schematic of Pavlovian conditioning that illustrates the formation of classical conditioned reflexes in dogs across 4 distinct stages (reproduced with permission from [77]; copyright 2024, American Chemical Society). In the “Memristor-based AI Systems” module, the mechanical flexibility of a 3D wearable neural network implemented via memristor synapses is demonstrated (reproduced under the terms of the CC BY 4.0 license [20]; copyright 2020, The Authors, published by Wiley); the module also displays an optoelectronic memristor array used for sensory computing and pattern recognition (reproduced with permission from [121]; copyright 2021, Elsevier), as well as a fully hardware-implemented ferroelectric memristor RC system (reproduced under the terms of the CC BY 4.0 license [116]; copyright 2023, The Authors, published by Springer Nature).

An overview of the evolution and integration of memristors within neural network computing is provided in Fig. 1, which schematically illustrates the interplay between memristive devices and key network modules. The structure of this review paper is organized as follows: History of Memristors and Neural Networks revisits the historical development and current status of memristor–neural network integration. Overview of Memristors systematically examines the architecture, fundamental principles of memristors, and their technological progress. Memristor-Based Artificial Neurons and Synapses elaborates on memristor-based implementations of artificial neurons and synapses, focusing on their bio-inspired operational mechanisms. Memristor-Enabled Neural Network Architectures provides a comprehensive taxonomy and architectural analysis of ANNs enabled by memristors, encompassing both classical and emerging paradigms. Neuromorphic Learning Implementation via Memristors systematically examines the concept of synaptic plasticity and its algorithmic realizations in memristive devices, addressing a broad spectrum of learning paradigms, including unsupervised, supervised, reinforcement, and associative learning. Memristor-Based Artificial Intelligence System Applications investigates system-level applications of memristors across AI domains, particularly highlighting optical, bio-inspired neuromorphic computing and flexible wearable devices’ implementations. Challenges and Outlook critically analyzes persistent challenges in memristor applications, proposing future research trajectories for next-generation brain-inspired chips and wearable systems, while outlining commercialization and industrialization prospects for memristor-integrated hardware neural networks.

## History of Memristors and Neural Networks

**Fig. 2.**
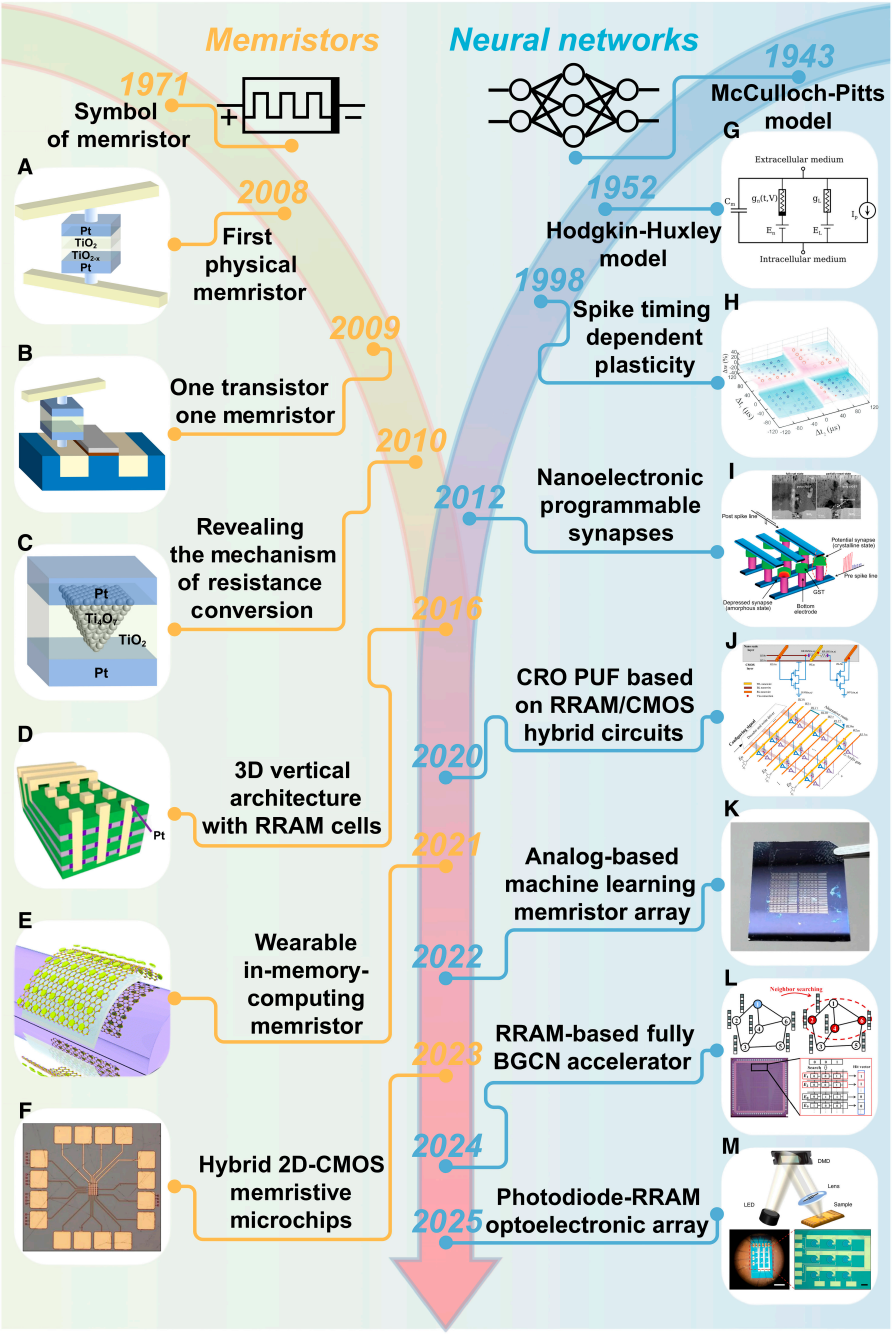
History of memristors and neural networks. (A) First physical memristor that reproduces Professor Chua’s theoretical postulates of resistive memory effects. (B) Invention of one-transistor-one-memristor architecture. (C) Detailed explanation of the mechanism of resistance conversion. (D) Invention of the 3D vertical architecture with RRAM cells. Reproduced under the terms of the CC BY 4.0 license [18]. Copyright 2016, The Authors, published by Springer Nature. (E) Development of the wearable in-memory computing memristor. Reproduced with permission from [131]. Copyright 2021, Royal Society of Chemistry. (F) Hybrid 2D-CMOS memristive microchips for in-memory computing. Reproduced under the terms of the CC BY 4.0 license [22]. Copyright 2023, The Authors, published by Springer Nature. (G) Memristive Hodgkin–Huxley circuit model. (H) Spike timing-dependent plasticity in second-order memristors. Reproduced with permission from [138]. Copyright 2017, Wiley. (I) Nanoelectronic programmable synapses. Reproduced with permission from [17]. Copyright 2012, American Chemical Society. (J) Lightweight CRO PUF based on RRAM/CMOS hybrid circuits. Reproduced under the terms of the CC BY 4.0 license [19]. Copyright 2020, The Authors, published by IEEE. (K) Analog-based machine learning memristor array. Reproduced under the terms of the CC BY 4.0 license [21]. Copyright 2022, The Authors, published by Springer Nature. (L) RRAM-based fully binarized graph convolutional network (BGCN) accelerator. Reproduced under the terms of the CC BY 4.0 license [23]. Copyright 2024, The Authors, published by Wiley. (M) Photodiode-RRAM optoelectronic array for in-sensor computing. Reproduced under the terms of the CC BY 4.0 license [24]. Copyright 2025, The Authors, published by Springer Nature.

The development of memristors and neural networks follows a continuous lineage, with both converging toward mutual prosperity through their integration. In this section, we review the developmental history of memristors and neural networks, thereby introducing an overview of those. In 1943, the neuron mathematical model established by McCulloch and Pitts [1] is commonly regarded as the origin of neural networks. In 1952, the Hodgkin–Huxley model described the dynamic relationship between neuronal membrane potential and ion channel gating using nonlinear differential equations [8], thereby providing the first molecular-level explanation for the generation and propagation of action potentials (Fig. 2G). However, since memristor theory did not exist during Hodgkin’s era, the 3 anomalous issues inherent in the model remained unresolved. The emergence of memristor theory provided a means to address these anomalies derived from empirical methods. (Professor Chua revised the model in 2014 using memristor theory [13].) In 1971, Professor Chua deduced the existence of the memristor based on the symmetry of passive components [11]; however, this theoretical development did not directly lead to the physical realization of memristors, and little progress was made throughout the 20th century. During this period, in 1998, Bi and Poo [14] conducted experimental studies on Hebbian rules, clarifying the quantitative impact of the timing differences between presynaptic and postsynaptic neuronal pulses on synaptic strength changes. The resulting spike timing-dependent plasticity (STDP) rule laid the foundation for brain-inspired computing and neuromorphic chip development (Fig. 2H). Subsequently, the so-called “winter” of memristor research was transformed in 2008 when Strukov and colleagues [12] at Hewlett-Packard Laboratories discovered and verified memristors exhibiting a pinched hysteresis loop by sandwiching an insulator between metals. This was the first instance of linking this resistive switching behavior to memristor theory, marking its transition from theory to practical application (Fig. 2A). Following this, in 2009, a 1T1R structure utilizing transistors to select memristor cells was developed, ingeniously mitigating leakage current issues while employing existing CMOS integration technology for system-level applications [15], thereby outlining future research directions (Fig. 2B). In 2010, explanations of the formation and rupture of metallic oxide filaments in memristor devices revealed the complete mechanism of resistive switching (Fig. 2C) [16]. The 2012 implementation of nanoelectronic programmable synapses (Fig. 2I) [17] and the 2016 realization of a 3D vertical RRAM cross-point array (Fig. 2D) laid the groundwork for the simulation of biological synapses using memristors and the integration of memristors with neuromorphic computing. At this juncture, the convergence of memristors and neural networks emerged as a prominent [18] research focus. In 2020, lightweight configurable ring oscillator (CRO) physical unclonable function (PUF) based on RRAM/CMOS hybrid circuits (Fig. 2J) [19], followed by wearable in-memory computing memristors in 2021 (Fig. 2E) [20], memristor arrays for machine learning-simulated dot product operations in 2022 (Fig. 2K) [21], hybrid 2-dimensional (2D)-CMOS memristive microchips in 2023 (Fig. 2F) [22], RRAM-based fully binarized graph convolutional network (BGCN) accelerator in 2024 (Fig. 2L) [23], and memristor sensor applications for Internet of Things (IoT) edge computing in 2025 (Fig. 2M) [24], have all been realized. The rapid evolution and boundless potential of the integration between memristors and neural networks is unmistakable.

## Overview of Memristors

### Fundamental principles of memristors

**Fig. 3.**
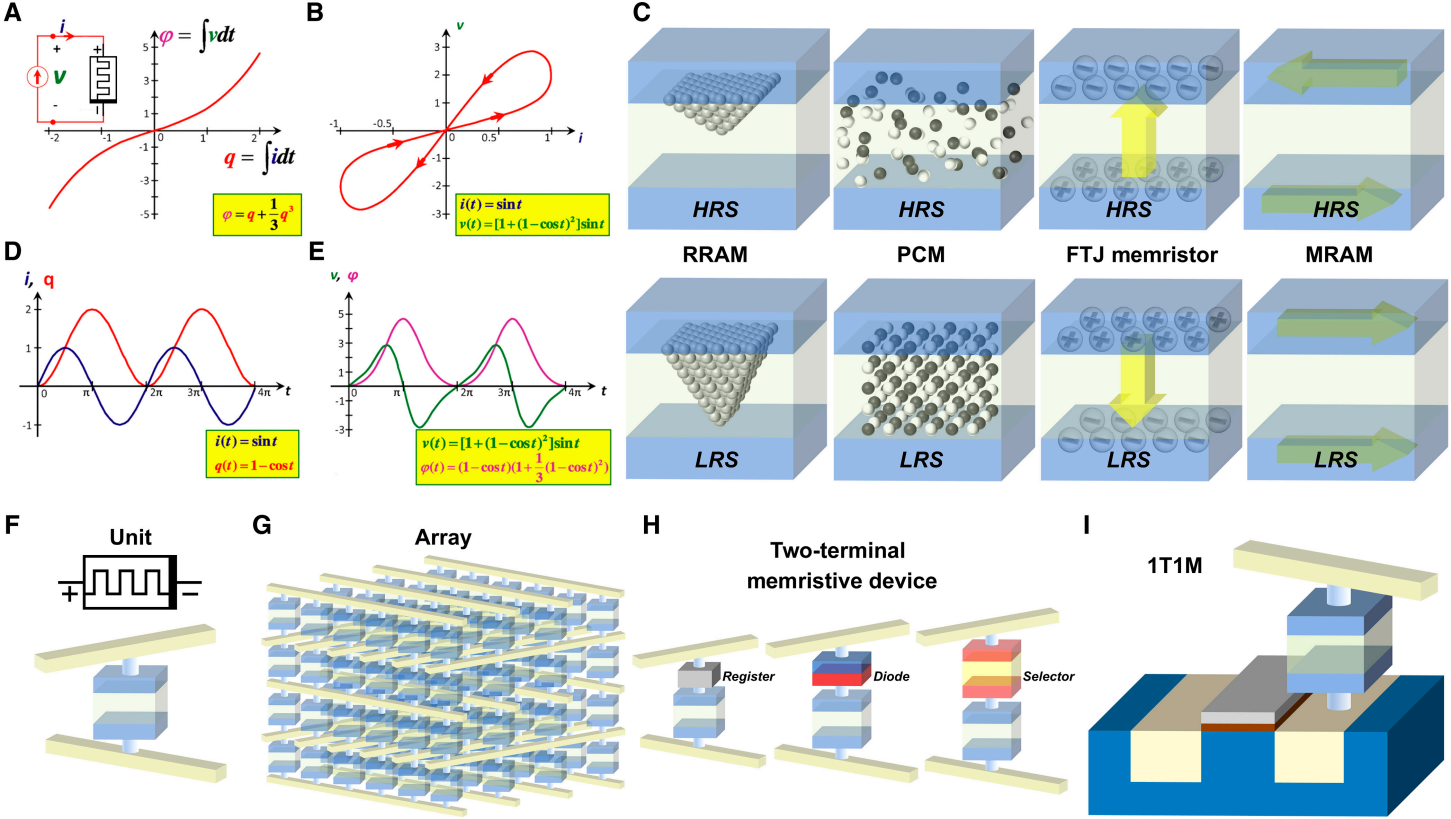
Fundamental principles and structures of memristors. (A) Memristor circuits and φ−q characteristic curve. Reproduced under the terms of the CC BY-NC 2.0 license [139]. Copyright 2011, The Author, published by Springer Nature. (B) Pinched hysteresis loop characteristic of memristors. Reproduced under the terms of the CC BY-NC 2.0 license [139]. Copyright 2011, The Author, published by Springer Nature. (C) Classification of resistive switching memories in a broad sense: RRAM, PCM, FTJ memristors, and MRAM, along with schematic diagrams of their structural changes between high and low resistance states. The light blue regions represent metal layers in the MIM structure, while the light yellowish green regions denote insulator layers. The conductive filament mechanism in RRAM is manifested as layer-by-layer atomic stacking along the electric field direction; the phase transition mechanism in PCM is manifested as mutual transformation between disordered amorphous and ordered crystalline structures; the ferroelectric polarization in FTJ memristors is manifested as directional switching of yellow arrows in the ferroelectric insulating layer under varying applied voltages; the magnetization reorientation in MRAM is manifested as current-dependent magnetic moment rotation of yellow arrows in the upper magnetized electrode layer. (D) Temporal relationships between current, electric charge, and time under applied sinusoidal current signals. Reproduced under the terms of the CC BY-NC 2.0 license [139]. Copyright 2011, The Author, published by Springer Nature. (E) Temporal relationships between voltage, magnetic flux, and time under applied sinusoidal current signals. Pinched hysteresis loop characteristic of memristors. Reproduced under the terms of the CC BY-NC 2.0 license [139]. Copyright 2011, The Author, published by Springer Nature. (F) Circuit symbol of a memristor and fundamental unit of its MIM structure. The pale yellow line-like structures vertically connected to the memristor structure correspond to the bit lines and word lines integrated within the crossbar array. (G) Self-rectifying memristor array. (H) Classification of 2-terminal memristive devices: 1R1M, 1D1M, and 1S1M, along with schematic diagrams of their structures. (I) Representative 3-terminal memristive device: 1T1M, along with its schematic diagram.

The memristor is an ideal 2-terminal circuit element characterized as a nonlinear resistor with memory functionality, denoted by the symbol shown in Fig. 3F. The memristor, first theorized by Professor Chua in 1971 as the fourth fundamental passive circuit element alongside the resistor, capacitor, and inductor, is a 2-terminal device whose instantaneous resistance depends on the integral of its past current–voltage history [11] (Fig. 3A). Mathematically, while a resistor obeys R=v/i, a memristor satisfies $\begin{array}{c} M=v\left(t\right)/i\left(t\right)=\left(\mathrm{d\varphi}/\mathrm{dt}\right)/\left(\mathrm{dq}/\mathrm{dt}\right)=\mathrm{d\varphi}/\mathrm{dq} \end{array}$, indicating that its resistance value is determined by the ratio of magnetic flux variation to charge variation. This implies that the memristor’s resistance depends on the historical relationship between magnetic flux and cumulative charge flow through the device, thereby functioning as a nonlinear resistive component with charge memory capability.

Memristors have been realized across diverse device platforms and, in 2015, were rigorously classified by Professor Chua into 4 categories: ideal, ideal generic, generalized, and extended memristors [25]. When driven by a periodic signal, they exhibit a characteristic pinched hysteresis loop in the current–voltage plane that progressively narrows at higher frequencies and approaches a linear resistor-like response (Fig. 3B, D, and E). Moreover, above specific voltage or current thresholds, memristors undergo reversible switching between high-resistance state (HRS) and low-resistance state (LRS), providing the basis for NVM functionality.

Regarding the macroscopic characteristics of memristors, Yang et al. [26] analyzed the computational performance requirements for memristor-based implementations. Specifically, for storage, key metrics include density, retention, and current–voltage nonlinearity; for memory, the focus is on reproducibility, endurance, reciprocal switching energy, and switching speed; and for neuromorphic applications, the number of states, ON/OFF current ratio, and OFF state resistance are critical. Memristors exhibit varying degrees of advantages across different characteristics depending on their target applications, demonstrating superior performance in properties critical to specific use cases.

### Physical architecture and functional arrays of memristors

The fundamental unit of a memristor is the metal–insulator–metal (MIM) structure, which consists of 2 metallic electrodes separated by an ultrathin insulating layer (Fig. 3F). Notably, not all MIM structures exhibit memristive effects; only those with an insulating layer demonstrating charge-dependent resistance modulation qualify as memristor building blocks. Based on resistance-switching mechanisms, generalized memristors can be categorized into 4 structural types (Fig. 3C). In detail, RRAM employs metal-oxide materials where oxygen vacancy migration leads to the formation and rupture of conductive filaments, thereby switching between HRS and LRS. Phase-change memory (PCM) utilizes phase-change materials in the insulating layer, achieving resistance switching through amorphous-to-crystalline phase transitions between high-resistance (amorphous) and low-resistance (crystalline) states. Ferroelectric tunnel junction (FTJ) devices, on the other hand, exploit the intrinsic polarization switching of ferroelectric materials; this polarization reversal modulates the tunneling resistance effect, enabling a reversible transition between distinct resistance states and thereby forming the basis of FTJ memristors. In a similar vein, magnetic tunnel junction (MTJ) devices modify the MIM configuration by replacing conventional electrodes with magnetized materials. The relative magnetization orientations of these layers determine the resistance state through the tunneling magnetoresistance effect, forming the foundation for magnetic random-access memory (MRAM).

The memristor model proposed by HP Labs in 2008 employs a $\mathrm{Pt}/\mathrm{Ti}O_{2-x}/\mathrm{Ti}O_{2}/\mathrm{Pt}$ structure [12], whose physical mechanism experimentally validates the theoretical model proposed by Professor Chua. The core of this device consists of a bilayer titanium oxide structure: a highly oxygen vacancy-doped ${\mathrm{TiO}}_{2-x}$ layer (LRS) and a lightly doped $\mathrm{Ti}O_{2}$ layer (HRS), forming an interfacial potential barrier. Under an applied electric field, oxygen vacancies migrate along the field direction, dynamically modulating the thickness of conductive channels and inducing reversible resistance switching [27]. This switching process maintains stable states after external stimuli removal, achieving nonvolatile data storage functionality, thereby providing both theoretical foundations and practical potential for applications in NVM devices and logic switches.

For memristors to be applied in practical systems, it is essential, just as with bipolar junction transistors (BJTs) and (metal-oxide-semiconductor field effect transistors) MOSFETs, to combine individual memristor units so that they function collectively. Memristor integration can be divided into passive array integration and active array integration. In passive array integration, only memristors and passive components are used to construct crossbar arrays by intersecting row and column lines, whereas active array integration typically involves connecting memristor units in series with transistors to form crossbar arrays. However, regardless of the integration method, an inevitable issue arises during data readout: the leakage current effect, where the current bypasses the HRS to flow through the LRS. In passive crossbar arrays, researchers have designed various 2-terminal memristor units (Fig. 3H), such as the 1R1M configuration, formed by connecting a resistor in series with a memristor; the 1D1M configuration, formed by connecting a diode in series with a memristor; and the 1S1M configuration, formed by connecting a selector (typically implemented using a bidirectional diode) in series with a memristor. In studies of the 1R1M structure, the resistor is used as a current limiter; this simple 2-terminal design is advantageous for low-cost applications. For the 1D1M/1S1M configurations, if RRAM memristors are employed, the resulting structure is referred to as 1D1R/1S1R. This array architecture, which utilizes a diode in series with a memristor unit, is characterized by a pronounced threshold effect and compact size, making it particularly suitable for applications that require higher array density and low power consumption, such as the fabrication of artificial neurons and synapses [28,29]. The RRAM compact model for 1S1R, studied by Chen et al. [30], provides a simulation foundation for the development of large-scale crossbar arrays. Similarly, the integration of tunnel selectors with memristors, studied by Upadhyay et al. [31], offers a solution for ensuring the material and electrical compatibility of 1S1R units. Currently, self-rectifying memristor arrays (Fig. 3G) represent another direction in passive array research. By constructing arrays with memristors that possess inherent self-rectifying effects, this approach offers lower circuit complexity, reduced power consumption, and enhanced scalability compared to architectures that incorporate additional devices. Research on self-rectifying memristors focuses on enhancing the rectification ratio and optimizing switching energy, with applications in storage class memory, in-memory computing, neuromorphic computing, and hardware security [32].

In active crossbar arrays, MOSFETs are typically connected in series with memristor units, with the MOSFET serving as a control switch to select array elements; this configuration is known as the 1T1M structure (Fig. 3I). The primary advantage of this structure is that it can be fabricated on-chip using existing integration processes, enabling system-level applications, such as memory devices [33] and logic circuits [34] that demand high reliability and stability of individual units. Compared to passive arrays, transistors can actively control selection via gate voltage adjustment, thereby enabling high-precision read and write operations. However, because transistor fabrication must be performed on a substrate, the 1T1M structure encounters challenges in 3D stacking and transistor miniaturization. Currently, research on 1T1M units is focused on 3D architectures and increasing array storage density. Yeh and Wong [35] investigated a one-transistor-N-RRAM (1TNR) array architecture and experimentally validated the characteristics of a 2D-integrated 1T4R chip. In this design, every four RRAM units share a common bottom electrode connected to the transistor’s drain, while their top electrodes are independently connected to other 1T4R units, forming a 64 × 64 array. By selecting RRAMs in the same row, leakage current is effectively suppressed. They also proposed a 3D-stacked 1T8R architecture, which lays the foundation for the design of multilayer memristor arrays. Furthermore, by integrating the advantages of passive arrays into active ones, using configuration word lines as natural selectors, analyzing physical layout design rules, and designing shared-drain configurations, Feng et al.’s [36] study on three-terminal RRAM crossbar arrays shows promise for applications in neural networks and edge computing. Assembling soft neuromorphic systems is also a crucial application domain for the pan-1T1M architecture. Kim et al. [37] fabricated a resistive pressure sensor using polymer semiconductors, mimicking biological afferent nerve behavior. By connecting it to an organic ring oscillator, they replaced the original fundamental 1T1R array and achieved low-power data processing through synaptic device configurations. Another crucial direction in active-matrix architectures is memtransistors. In such structures, the gate dielectric layer of MOSFETs is typically either overlaid with a memristive insulating material or replaced by such materials, enabling programmable conductance controlled by gate voltage and drain–source voltage pulses. This design allows the device to simultaneously exhibit field-effect modulation capability and NVM functionality. Studies by Dodda et al. [38], who developed a programmable optoelectronic transistor using monolayer MoS_2_, and Schranghamer et al. [39], who constructed a non-volatile multi-state resistive memory based on graphene, collectively demonstrate that memtransistors are evolving into key in-memory computing devices for advancing sensor-in-computing architectures and neural network implementations. ​In Table, we summarize the memristor architectures discussed in this section and provide a systematic comparison of their ​core metrics, serving as a benchmark for future research.

**Table.** Comparison of performance metrics of memristors with different mechanisms

Classification	Current on/off ratios	Endurance (cycles)	Retention (years)	Area (F^2^)	Switching speed (ns)	Energy consumption (fJ)
RRAM	$10^{2}-10^{6}$	$10^{6}-10^{9}$	>10	4−30	$<10^{2}$	$1-10^{2}$
PCM	$10-10^{3}$	$10^{7}-10^{9}$	>10	4−8	$10-10^{4}$	$10^{2}-10^{4}$
FTJ memristor	$10^{2}-10^{3}$	$10^{8}-10^{9}$	$>10^{-2}$	4−20	$10-10^{2}$	$1-10^{2}$
MRAM	1−10	$10^{6}-10^{15}$	>10	4−30	$<10^{2}$	$1-10^{2}$
Memtransistor	$10^{2}-10^{3}$	$10^{4}-10^{6}$	$10^{-2}-10$	4−30	$10^{2}-10^{5}$	$10-10^{5}$

### Recent advances in memristor materials

**Fig. 4.**
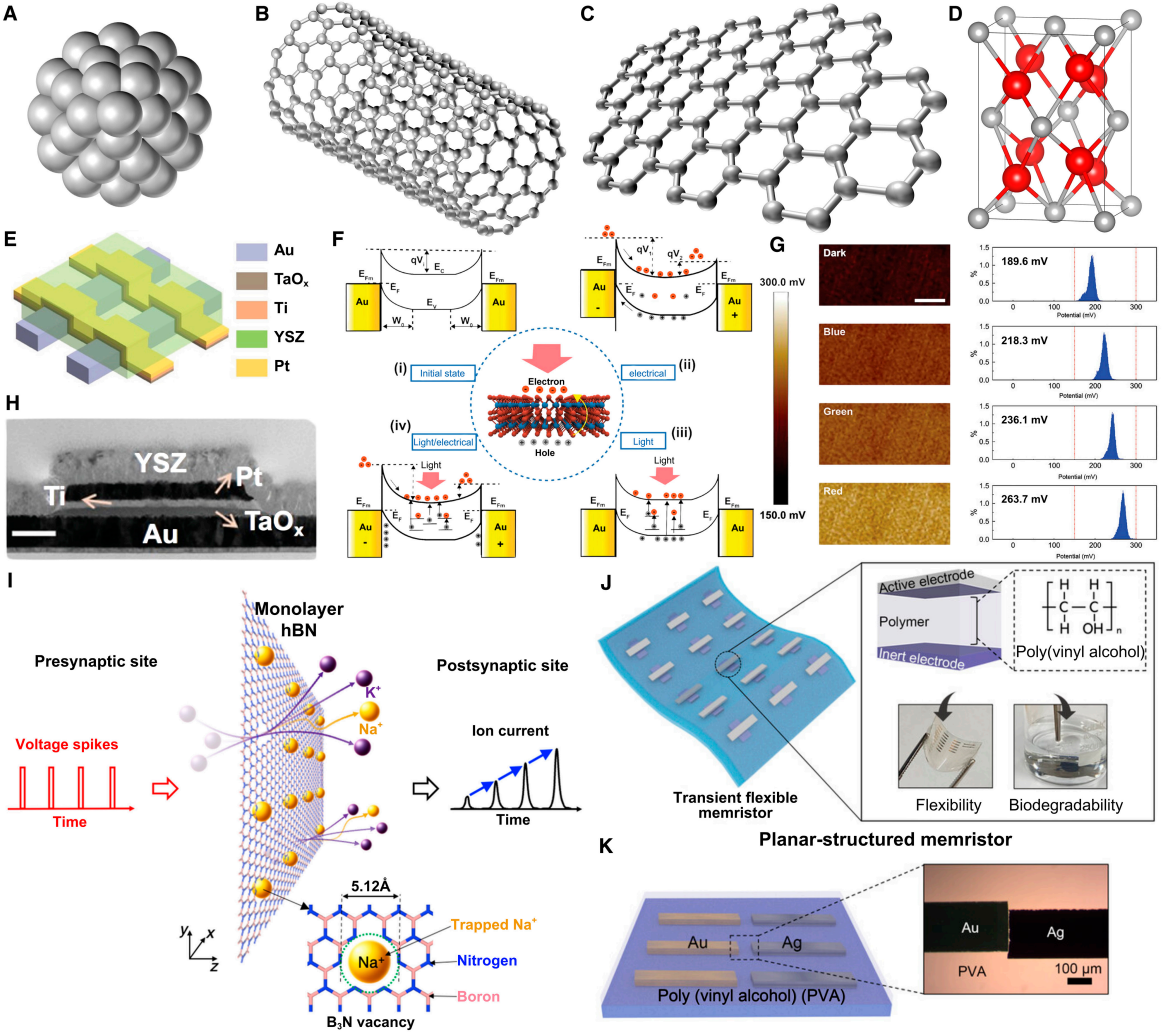
Recent advances in memristor materials. (A) Schematic diagram of 0D quantum dot memristor materials. (B) Schematic diagram of 1D memristor materials. (C) Schematic diagram of 2D memristor materials. (D) Schematic diagram of metal oxide memristor materials (tetragonal $\mathrm{Hf}O_{2}$). (E) Structural configuration of a $\mathrm{Pt}/\mathrm{Ti}/\mathrm{Ta}O_{x}/\mathrm{Au}$ memristor device. Reproduced with permission from [51]. Copyright 2021, Wiley. (F) 2D retinal-morphic device based on $WS_{2}$ memristor array. Reproduced with permission from [59]. Copyright 2024, Wiley. (G) Photo-modulated performance evolution of the device. Reproduced with permission from [59]. Copyright 2024, Wiley. (H) Cross-sectional transmission electron microscopy image of the memristor. Reproduced with permission from [51]. Copyright 2021, Wiley. (I) 2D nanofluidic memristor involving bicationic ion transport driven. Reproduced under the terms of the CC BY 4.0 license [50]. Copyright 2024, The Authors, published by American Association for the Advancement of Science. (J) Simulated structure of polyvinyl alcohol (PVA)-based electrochemical metallization memristor unit with mechanical flexibility and biodegradability. Reproduced under the terms of the CC BY 4.0 license [61]. Copyright 2023, The Authors, published by Wiley. (K) Simulated structure and microscopic configuration of PVA-based electrochemical metallization memristor. Reproduced under the terms of the CC BY 4.0 license [61]. Copyright 2023, The Authors, published by Wiley.

Because of the unique properties of the memristor’s MIM structure, more researchers have extensive research interests in the memristor insulating layer materials, but studies on electrode materials remain relatively limited. Figure 4 presents the latest advancements in memristor material implementations. The development of electrode materials has demonstrated a diversified trend [40]. In early stages, inert metal electrodes (e.g., Au and Pt) were predominantly employed as conductive media [41]. While their chemical stability ensures device reliability, they cannot actively participate in resistive switching reactions. With in-depth investigations into electrochemical metallization mechanisms, active metal electrodes (e.g., Cu and Ag) have been widely adopted. The migration of metal ions facilitates conductive filament formation, significantly enhancing the controllability of resistive switching and switching speed [42]. Recent years have witnessed growing research interest in carbon-based materials (e.g., graphene [43] and carbon nanotubes [44]) due to their high conductivity and atomic-level thickness. Graphene electrodes not only effectively mitigate failure mechanisms caused by ion penetration but also enable the fabrication of flexible devices. Furthermore, nitride electrodes (e.g., TiN and TaN) exhibit substantial potential for large-scale integration in circuits due to their CMOS process compatibility. Meanwhile, the introduction of transparent oxide electrodes (e.g., ITO and FTO) has accelerated advancements in optoelectronic memristors and flexible transparent devices.

The application development of memristors in resistive switching materials has become increasingly diverse. Traditionally, metal oxides serve as the insulating layer in MIM devices, where mechanisms such as oxygen vacancy migration and the formation and rupture of conductive filaments determine resistive switching. However, emerging materials, including 0D quantum dots, 1D nanowires, 2D materials, as well as 3D inorganic and organic compounds, are also being explored as alternative insulating layers. Figure 4A to D categorizes memristor materials based on their dimensional characteristics. 0D quantum dot memristor materials achieve resistive switching by leveraging phenomena such as quantum confinement-induced discrete energy levels, localized charge transport, and interfacial Schottky barriers [45,46]. 1D memristor materials, characterized by nanowire structures, realize directional conduction and the formation of conductive filaments through anisotropic conductive channels [47]. 2D memristor materials (e.g., Graphdiyne and hexagonal boron nitride) modulate switching behavior by exploiting their atomically thin structures and large surface areas, demonstrating superior performance in heterogeneous integration compatibility and mechanical flexibility [48]. Graphdiyne leverages its triangular pore structure and sp-hybridized carbon atoms to exhibit low ion diffusion barriers and strong ion adsorption capabilities, enabling its direct use in constructing an active insulating layer that mimics synaptic cleft-like information transmission [49]. Noh and Smolyanitsky [50] developed a monolayer hexagonal boron nitride memristor film (Fig. 4I), unveiling the transport mechanisms of aqueous ions in subnanoporous materials at the molecular level. Binary metal oxides (e.g., ${\mathrm{TiO}}_{x}$, ${\mathrm{HfO}}_{x}$ (Fig. 4D), ${\mathrm{AlO}}_{x}$, ${\mathrm{TaO}}_{x}$, ${\mathrm{ZrO}}_{x}$, ${\mathrm{CuO}}_{x}$, ${\mathrm{WO}}_{x}$) have been widely utilized in memristors due to their superior resistive switching characteristics and process maturity, with their switching mechanisms primarily attributed to the formation and annihilation of oxygen vacancies [41]. Cheng et al. developed a $\mathrm{Pt}/\mathrm{Ti}/{\mathrm{TaO}}_{x}/\mathrm{Au}$-structured memristor (Fig. 4E, H) that emulates astrocyte functionality, demonstrating potential applications in neuromorphic computing [51]. Lin et al. constructed a 3D circuit composed of 8-layer monolithically integrated memristors, achieving precise parallel edge detection in video imagery [52]. Perovskite-structured oxides (e.g., $\text{SrTi}O_{3}$, organic-inorganic hybrid perovskites) leverage their unique crystal configurations and charge-coupling effects to enable synaptic behavior simulation, offering novel approaches for neuromorphic computing [53,54]. However, challenges in doping control and CMOS process incompatibility result in overall inferior device metrics compared to binary metal oxides, limiting their application prospects. Organometal halide perovskite-based synapses, by uniquely integrating low ion migration barriers, tunable optoelectronic responses, and solution-processable fabrication, achieve energy-efficient synaptic plasticity, not only bridging the performance gap with binary oxide materials but also expanding their application prospects in flexible bioinspired robotics and biological systems [55–57]. Perovskite oxide materials can also form memtransistors. Sen et al. demonstrated hetero-stacked memtransistors compatible with CMOS technology by employing $\text{SrTi}O_{3}$ as the gate dielectric. Leveraging their cryogenic memory properties, this approach addresses the demand for CMOS-compatible solutions in in-memory computing and neuromorphic architectures [58]. Solid-state electrolyte materials exhibit ion channels formed by crystalline defects, enabling resistive switching through conductive filament formation/rupture. These materials support multilevel storage, logical operations, and synaptic emulation, making them ideal for developing low-power, high-endurance memristive devices. Among solid-state electrolytes, chalcogenide semiconductors (e.g., ${\mathrm{Ag}}_{2}S$, ${\mathrm{WS}}_{2}$, ${\mathrm{MoS}}_{2}$) have emerged as promising candidates for next-generation memristors due to their layered structures and exceptional mechanical flexibility, which facilitate high-speed switching and energy-efficient operation. Gong et al. demonstrated a ${\mathrm{WS}}_{2}$-based memristor array implementing 2D retinal morphology (Fig. 4F, G), enabling light-modulated visual recognition [59]. Organic solid-state electrolytes (e.g., PMMA, PVA, $P_{3}\mathrm{HT}$) present another promising avenue, demonstrating broad application potential in memristors through their low-cost fabrication, low-temperature processability, and flexible and stretchable characteristics, thereby enabling novel device options for wearable electronics and bioelectronic systems. Xu et al. [37,60] employed organic nanowire synaptic transistors to mimic the morphology of nerve fibers. Leveraging their slender geometry, flexibility, and scalability, they constructed bioinspired synapses with femtojoule-level energy consumption per synaptic event. Oh et al. [61] developed a polyvinyl alcohol-based electrochemical metallization memristor (Fig. 4J and K), representing an environmentally friendly wearable device platform.

## Memristor-Based Artificial Neurons and Synapses

### Artificial neurons

**Fig. 5.**
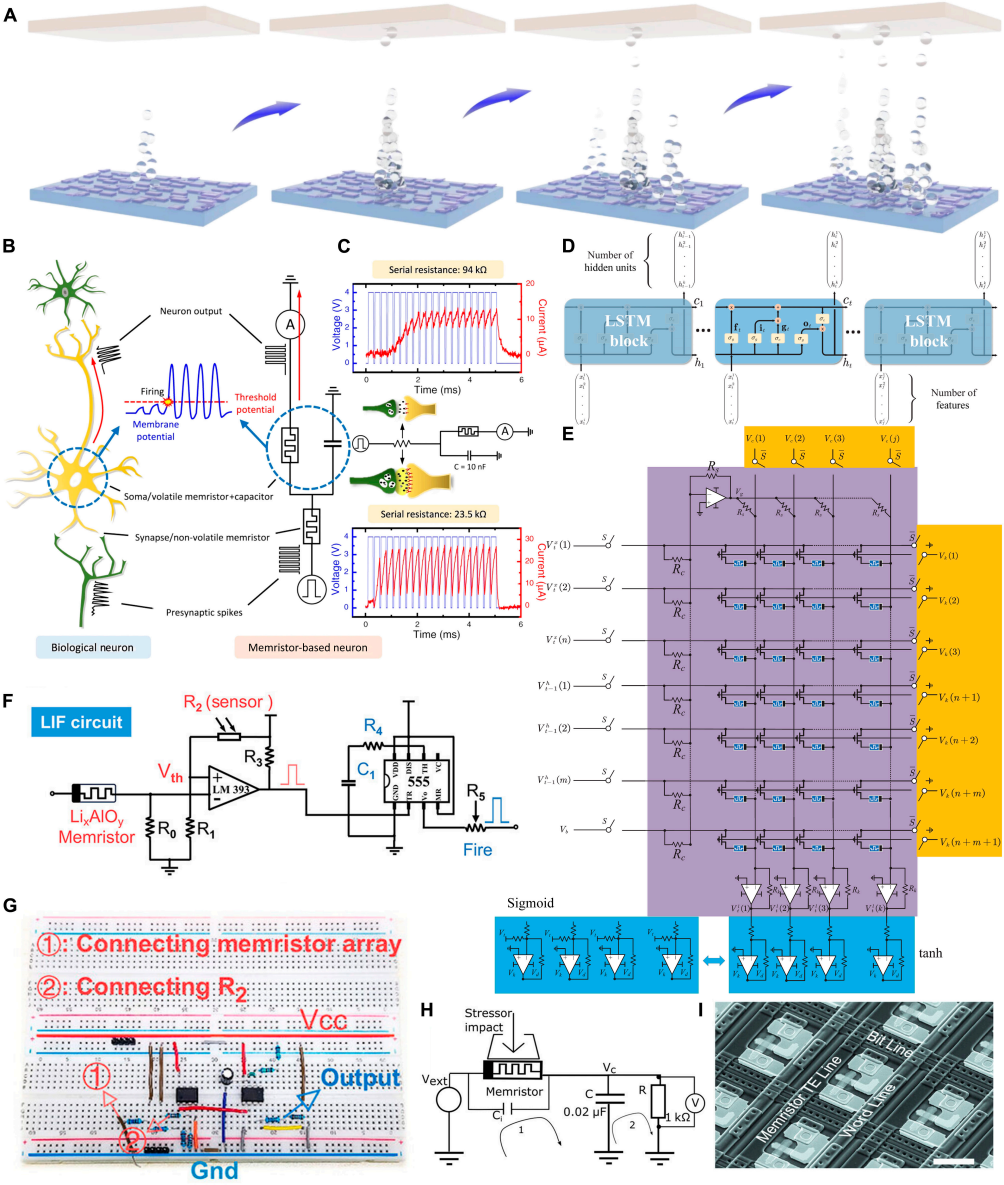
Artificial neuron implementations. (A) Schematic of $\mathrm{Ag}/\mathrm{Mo}S_{2}/\text{HfAl}O_{x}$ memristor-based neuron model with pulsed stimulation mechanism. Reproduced under the terms of the CC BY 4.0 license [44]. Copyright 2022, The Authors, published by Springer Nature. (B) Comparative illustration of biological neurons and gradual $\mathrm{Ti}O_{x}$ memristor-based LIF neurons. Reproduced under the terms of the CC BY 4.0 license [69]. Copyright 2022, The Authors, published by Springer Nature. (C) Output responses of $\mathrm{Ti}O_{x}$ memristor-based LIF neurons under varying synaptic strengths. Reproduced under the terms of the CC BY 4.0 license [69]. Copyright 2022, The Authors, published by Springer Nature. (D) Diagram of a standard LSTM cell. Reproduced under the terms of the CC BY 4.0 license [65]. Copyright 2023, The Authors, published by World Scientific Publishing Company. (E) Circuit diagram of the memristor-based LSTM unit. Reproduced under the terms of the CC BY 4.0 license [65]. Copyright 2023, The Authors, published by World Scientific Publishing Company. (F) Threshold-tunable LIF neuron circuit based on $Li_{x}\mathrm{Al}O_{y}$ memristors with peripheral circuitry. Reproduced with permission from [70]. Copyright 2024, American Chemical Society. (G) Photograph of the threshold-tunable LIF neuron circuit. Reproduced with permission from [70]. Copyright 2024, American Chemical Society. (H) $\mathrm{Si}O_{x}/\mathrm{Ag}$ memristor-based neuron circuit for pressure sensing applications. Reproduced under the terms of the CC BY 4.0 license [71]. Copyright 2024, The Authors, published by Springer Nature. (I) SEM image of 1T1R architecture for RC implementation. Reproduced under the terms of the CC BY 4.0 license [67]. Copyright 2019, The Authors, published by Wiley.

Artificial neurons constitute the fundamental computational units of neural networks, designed to emulate partial biological neuronal characteristics. Different types of ANNs employ various artificial neuron models to accommodate their specific computational requirements. CNNs typically use the rectified linear unit (ReLU) as their activation function to achieve sparse activation [62] and to enhance training stability and computational efficiency. In contrast, RNNs usually adopt the sigmoid or tanh function as their activation function [63] to maintain the flow of information across time steps, given their need to manage long-term dependencies. However, RNNs are susceptible to the vanishing gradient problem when handling long-term dependencies. Consequently, LSTM units and gated recurrent units have been introduced to leverage gating mechanisms for more effective retention and updating of state information [64] (Fig. 5D). As illustrated in Fig. 5E, Dou et al. [65] tuned amplifier parameters to modify the activation function and leveraged a memristor crossbar array to accelerate matrix-vector multiplications during backpropagation; their LSTM unit thereby emulated the behavior of a sigmoid activation function. Similarly, Li et al. [66] designed a 1T1R crossbar array that accelerates LSTM network operations. To reduce computational cost and focus on hardware characteristic development, only the readout layer weights of the RNN are trained, and reservoir computing (RC) is widely employed to evaluate memristor-based neurons. Midya et al. [67] developed an RC system through a 1T1R structure combining graded $\mathrm{Ti}O_{x}$ memristors with transistors, serving as fundamental neurons in the RNN readout layer. This architecture significantly reduces training complexity. However, as illustrated in Fig. 5I, at the device level, these implementations utilize 1T1R crossbar units to realize in-memory computing arrays. Various artificial neuron models, through diverse activation mechanisms and structural designs tailored to the specific requirements of their respective networks, exhibit unique advantages and limitations, thereby providing a range of design strategies and theoretical support for memristor-based neuron implementations.

Broadly speaking, activation functions are regarded as the core computational elements of traditional artificial neurons, whereas the memristor-based implementations of spiking neurons, an emerging paradigm in neuromorphic computing, have only recently begun to gain traction over the past decade [68]. Building on the aforementioned analysis of conventional artificial neuron models, memristors not only retain information when powered off due to their nonvolatile nature but also emulate key biological neuron characteristics, including excitability, inhibitory function, and integrate-and-fire behavior, through dynamic modulation of their resistance states. SNNs incorporate the leaky integrate-and-fire (LIF) neuron model, which emulates the action potential firing mechanism of biological neurons. This enables SNNs to process temporally dependent signals in an event-driven manner and to exhibit significant advantages in energy efficiency. As demonstrated in Fig. 5A, Wang et al. [44] implemented an integrate-and-fire mechanism in artificial neurons using $\mathrm{Ag}/\mathrm{Mo}S_{2}/\text{HfAl}O_{x}$ memristors. Under pulsed stimulation, the memristor’s conductance progressively accumulates until reaching a threshold, triggering voltage spikes analogous to biological action potentials. As shown in Fig. 5B and C, Park et al. [69] implemented a $\mathrm{Ti}O_{x}$ memristor crossbar array to construct LIF neurons. Their design features a transistor-free, capacitor-parallel architecture that enables individual memristor access to implement LIF neurons, with the firing-pulse amplitude modulated by the presynaptic spike intensity. Recently, Ke et al. [70] innovatively developed a threshold-tunable LIF neuron capable of integrating pulsed inputs from upstream memristor synapses, achieving both integration and spiking through efficient signal processing and adjustable thresholds (Fig. 5F and G). Additionally, memristor neurons can be physically stimulated to emit spike pulses for pressure sensing; Pattnaik et al. demonstrated $\mathrm{Si}O_{x}/\mathrm{Ag}$ memristor neurons for testing electrical signals and mechanical impact responses [71]. Figure 5H shows the neuron circuit used in this work. Overall, memristors, by virtue of their nonvolatility and dynamic modulation capabilities, not only replicate the excitation, inhibition, and integrate-and-fire functions of biological neurons but also open new research avenues for the development of efficient, low-power neural network hardware.

### Artificial synapses

**Fig. 6.**
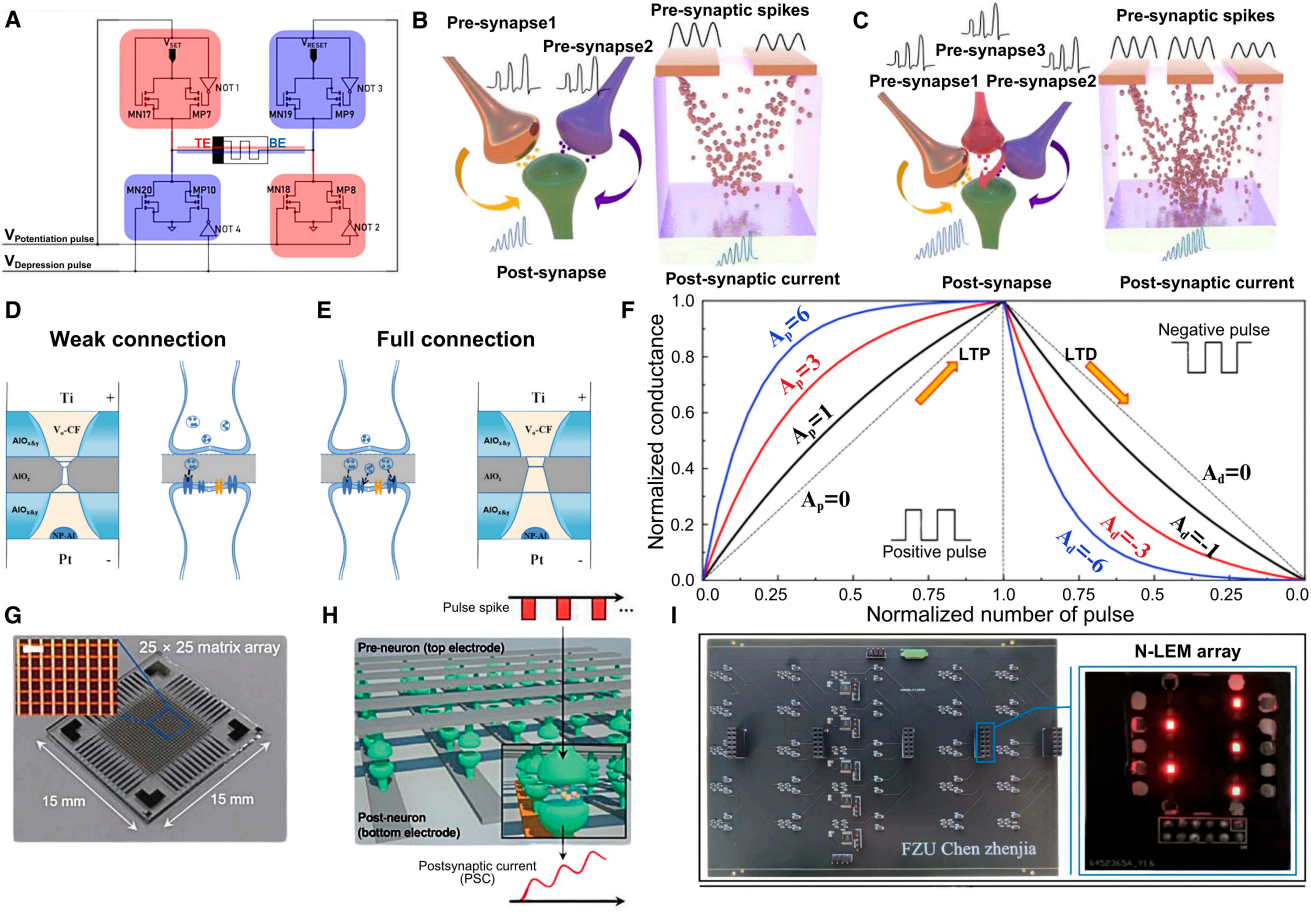
Artificial synapse implementations. (A) Hybrid CMOS–memristor H-bridge synapse circuits. Reproduced under the terms of the CC BY-NC-ND 4.0 license [75]. Copyright 2024, The Authors, published by Springer Nature. (B) Dual presynaptic spike integration mechanism with single postsynaptic output. Reproduced with permission from [77]. Copyright 2024, American Chemical Society. (C) Triple presynaptic spike coordination model with single postsynaptic output. Reproduced with permission from [77]. Copyright 2024, American Chemical Society. (D) Weak filament connection state in $\mathrm{Al}O_{x}$ memristor. Reproduced with permission from [78]. Copyright 2024, Wiley. (E) Robust filament connection state in $\mathrm{Al}O_{x}$ memristor. Reproduced with permission from [78]. Copyright 2024, Wiley. (F) Conductance–pulse characteristics demonstrating LTP/LTD in memristor array for CNN matrix multiplication. Reproduced under the terms of the CC BY 4.0 license [79]. Copyright 2021, The Authors, published by Wiley. (G) Optical microscopy image of $\mathrm{Ti}O_{x}$ memristor crossbar array. Reproduced under the terms of the CC BY 4.0 license [74]. Copyright 2022, The Authors, published by Wiley. (H) Synaptic microstructure within a $\mathrm{Ti}O_{x}$ memristor array. Reproduced under the terms of the CC BY 4.0 license [74]. Copyright 2022, The Authors, published by Wiley. (I) Hardware implementation of synaptic connections between hidden layers using a light-emitting memristor. Reproduced under the terms of the CC BY 4.0 license [76]. Copyright 2024, The Authors, published by Springer Nature.

Artificial synapses, designed to mimic inter-neuronal signaling in biological systems, achieve dynamic synaptic weight modulation by utilizing MIM memory cells to emulate the dynamic modulation characteristics of biological synapses [72]. As previously detailed, MIM-based memory architectures, including RRAM, PCM, FTJ memristor, and MRAM, demonstrate reproducible resistance switching between HRS and LRS under controlled conditions, enabling biomimetic synaptic plasticity emulation through analog conductance tuning [73]. For instance, Jang et al. demonstrated a $\mathrm{Ti}O_{x}$ memristor array serving as artificial synaptic connections between pre-neurons and post-neurons. As shown in Fig. 6G and H, repetitive pulsed stimulation induces graded post-synaptic current enhancement through conductance potentiation, achieving STDP learning rules [74]. Furthermore, hybrid CMOS–memristor circuits (Fig. 6A) implement analog memory [75] and energy-efficient analog dot-product operations in ANNs. Memristive devices implement configurable analog weights, crossbar wires act as axons and dendrites, and CMOS serves as a summing amplifier. Input voltages on memristors are multiplied by their conductances and then summed by the CMOS amplifier, realizing a key operation for ANNs [26]. Novel optoelectronic memristive devices (Fig. 6I) enable optical cross-layer transmission strategies. When constructing hidden synaptic layers, these devices achieve an accuracy in the fully connected network (FCN) that is comparable to that of electrical-only implementations, approaching the accuracy level of ideal transmission in FCNs, which provides a new avenue for low-energy and high-parallel neural network implementation [76].

In biological neural networks (BioNNs), axonal convergence from multiple presynaptic neurons collectively modulates postsynaptic responses through cooperative signaling mechanisms. As illustrated in Fig. 6B and C, Meng et al. [77] developed a polyethylene terephthalate/copper ion array device that mimics “dual-axonal cooperation” and “tri-axonal cooperation” phenomena observed in vivo. This neuromorphic system achieves enhanced synaptic weight updates through copper ion transport dynamics, which exhibit analogous characteristics to calcium diffusion in biological synapses. Inspired by neurotransmitter release across synaptic clefts, Yue et al. [78] engineered a quintuple-layered $\mathrm{Al}O_{x}$ memristor with a V-shaped oxygen vacancy gradient (minimum oxygen concentration at the central layer). As shown in Fig. 6D and E, this architecture emulates synaptic cleft functionality through 2 distinct conductive states: weak filament connection (partial ion channel opening) and robust filament formation (full channel activation), demonstrating biomimetic adaptability for BioNNs.

Memristive synaptic devices exhibit graded long-term potentiation (LTP) and long-term depression (LTD) under varying pulse parameters, as demonstrated by their tunable conductance states in Fig. 6F [79]. This graded plasticity mechanism, governed by pulse amplitude-dependent ion migration dynamics and filament formation kinetics, will be systematically analyzed in Neuromorphic Learning Implementation via Memristors through quantitative modeling of STDP rules. These device-level dynamics form the cornerstone for constructing neuromorphic networks. In Memristor-Enabled Neural Network Architectures, we investigate how to construct complete neural networks using memristive neurons and synapses from the perspective of system-scale architectures, and explore their methods for processing spatiotemporal signals, thereby establishing the neuromorphic device foundation for efficient hardware learning algorithms.

## Memristor-Enabled Neural Network Architectures

ANNs are computational models emulating BioNNs, originating from the McCulloch–Pitts neuron model (1943) [1]. Key milestones include Hebbian learning theory (1940s) for synaptic weight adaptation [80], Rosenblatt’s Perceptron (layered input-hidden-output structure) [2,81], and Hopfield networks (bidirectional symmetric connections) [3].

**Fig. 7.**
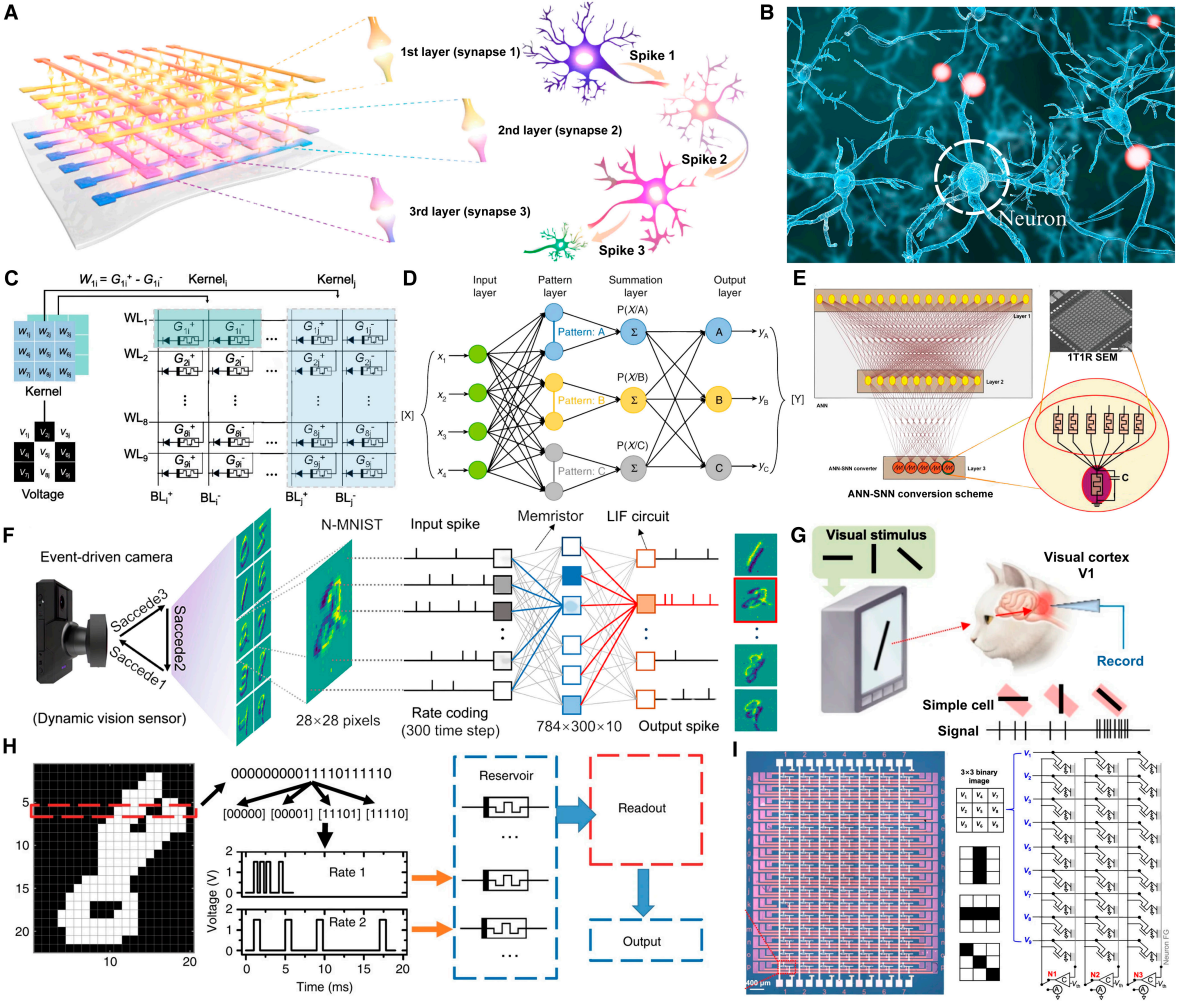
Taxonomy of ANN architectures. (A) Bio-inspired ANN device implemented via memristors demonstrating structural analogy with BioNN. Reproduced with permission from [82]. Copyright 2020, American Chemical Society. (B) Central nervous system illustrating neuronal–glial networks with synaptic signal transmission. (C) Mapping relationship between convolutional kernels and 1S1R arrays in CNN computation using 1S1R arrays. Reproduced under the terms of the CC BY-NC-ND 4.0 license [119]. Copyright 2025, The Authors, published by Springer Nature. (D) PNN architecture with pattern layer (Gaussian kernel estimation) and summation layer (Bayesian classification). Reproduced under the terms of the CC BY 4.0 license [90]. Copyright 2019, The Authors, published by Springer Nature. (E) ANN-to-SNN conversion framework employing threshold balancing and temporal encoding. Reproduced with permission from [100]. Copyright 2019, Wiley. (F) SNN circuit for dynamic visual recognition implemented with a threshold-tunable LIF neuron constructed from $Li_{x}\mathrm{Al}O_{y}$ memristors. Reproduced with permission from [70]. Copyright 2024, American Chemical Society. (G) Temporal coding scheme for SNN utilizing STDP. Reproduced under the terms of the CC BY 4.0 license [97]. Copyright 2023, The Authors, published by Springer Nature. (H) Flowchart of memristor-based RC system for digit recognition. Reproduced under the terms of the CC BY 4.0 license [95]. Copyright 2017, The Authors, published by Springer Nature. (I) Photograph and circuit schematic of an SNN based on a $VO_{2}$-based LIF neuron model. Reproduced under the terms of the CC BY 4.0 license [97]. Copyright 2023, The Authors, published by Springer Nature.

BioNNs, exemplified by the central nervous system (Fig. 7B), feature neurons with dendrites (short branched inputs) and axons (long outputs), interconnected via synapses for signal transmission, supported by glial cells in metabolic regulation. A 3D ANN implemented using a 3-layer stacked $\mathrm{Pt}/\text{HfAl}O_{x}/\mathrm{TaN}$ memristor (Fig. 7A) demonstrates the potential of 3D crossbar architectures for low-power neuromorphic computing. This system achieves high-accuracy image recognition by emulating tertiary synaptic connections between neurons, underscoring the promising trajectory of bio-inspired ANN development [82].

### CNNs

A CNN is a feedforward neural network architecture designed for processing grid-structured data through convolution kernels. Inspired by biological perception mechanisms, it is widely applied in tasks such as image classification and object detection. A standard CNN architecture comprises an input layer, hidden layers (including convolutional layers, pooling layers, and fully connected layers), and an output layer. As illustrated in Fig. 7C, the 2D convolution operation in convolutional layers involves spatial repetition of 3 × 3 kernels across local pixel regions [90]. Each kernel computes dot products between its weights and local input values, enabling feature extraction. Subsequently, extracted features undergo dimensionality reduction through pooling layers before being integrated in fully connected layers for final classification.

Building on this architecture, memristor crossbar arrays provide a promising hardware-level implementation for both convolutional and fully connected layers. In convolutional layers, kernel weights are encoded as conductance values within the memristor array, enabling efficient local vector–matrix multiplications when input voltages are applied. For fully connected layers, the entire synaptic weight matrix can similarly be mapped onto the crossbar, allowing for highly parallel in situ computation with minimal latency and power consumption. This memristor-based analog convolution operation, CNN recognition circuit, was first proposed by Yakopcic et al. [83,84].

However, as the number of kernels and connections scales up, traditional 2D array architectures face challenges in density and routing efficiency, motivating the exploration of more compact 3D integration schemes [85,86]. To achieve parallel processing of multiple kernels in hardware implementation, Lin et al. [52] innovatively extended 2D array designs to 3D configurations. By densely integrating kernels within a single 3D memristor array, they demonstrated high-density CNN computation under spatial constraints. This breakthrough highlights the potential of 3D integrated technology for enabling energy-efficient neuromorphic systems through memristor-based convolutional operations. An alternative approach involves optimizing the neural network architecture itself. For instance, Guo and colleagues [87,88] proposed a pruning and quantization algorithm to eliminate redundant synaptic weights and reduce inter-neuronal connectivity. By normalizing the input data into a constrained range and strategically minimizing the number of required memristor arrays, their method achieves high recognition speed while significantly lowering power consumption.

Similarly, a feedforward network that replaces conventional hidden layers with a separate pattern layer and summation layer to implement Bayes’ classification is termed a probabilistic neural network (PNN) [89]. As shown in Fig. 7D, the input layer transmits data features to the pattern layer, where each neuron estimates the probability density function (PDF) of training samples using Gaussian kernel functions. The summation layer aggregates these PDF estimates across categories, enabling the output layer to execute classification decisions through comparative evaluation of posterior probabilities [90]. Recent research has focused on overcoming the hardware complexity of Gaussian kernel evaluations and mitigating sneak-path currents in large-scale memristor crossbar arrays [91,92], resulting in variational inference training and modular array partitioning schemes that enable real-time probabilistic inference with significantly reduced energy consumption and enhanced throughput.​

### RNNs

An RNN is a class of neural networks characterized by internal cyclic connections, designed for processing sequential data such as time series, natural language, and speech signals. Its architecture typically comprises an input layer, hidden layer with recurrent units, and output layer. The recurrent units, central to RNN’s functionality, enable parameter sharing across time steps while maintaining temporal dependencies through hidden state propagation. The fundamental architecture demonstrates how sequential inputs are processed through identical recurrent units at each time step. The hidden state $h_{t}$ is updated by combining previous state $h_{t-1}$ with current input $x_{t}$, following the equation $h_{t}=\sigma \left(W_{h}h_{t-1}+W_{x}x_{t}+b\right)$, where σ denotes the activation function. This recurrent mechanism enables temporal pattern modeling through continuous information flow [64,66]. As discussed in Memristor-Based Artificial Neurons and Synapses, the LSTM cell comprises a structure that integrates the forgetting gate, input gate, and output gate. In contrast, the LSTM unit serves as a parameterized module within the cell. Li et al. [66] proposed a hardware acceleration solution based on a memristor-applied LSTM cell architecture, which alleviates the limitations of the LSTM network imposed by the von Neumann architecture to some extent.

RC constitutes a computational framework derived from RNNs, characterized by generating rich dynamic responses through randomly connected RNNs with fixed weights. Its core principle involves training only the output layer weights to accomplish specific tasks through linear regression optimization [93]. A canonical RC architecture comprises 3 components: (a) an input layer that projects signals into a high-dimensional reservoir via fixed-weight matrices, (b) a reservoir (dynamic system) where recurrent connections generate transient states through nonlinear activation functions (e.g., tanh and sigmoid), and (c) an output layer that linearly combines reservoir states for prediction. This design leverages the “echo state property” to ensure fading memory of past inputs. Echo state networks (ESNs) [94] represent a quintessential RC implementation where the reservoir’s sparse connectivity and spectral radius are fixed to maintain dynamical stability. Unlike conventional RNNs requiring backpropagation through time, ESNs solely train output weights using ridge regression, reducing computational complexity by orders of magnitude. This enables efficient applications in chaotic time series prediction and real-time signal processing tasks like speech recognition. Figure 7H illustrates the workflow of handwritten digit recognition utilizing memristor short-term ionic dynamics to construct the RC system [95]. Milano et al. [96] developed a self-organized nanowire network to establish stochastic connectivity among multiple nonlinear memristive elements. By leveraging the nonlinear dynamics and fading memory properties of nanowire memristors, they mapped the evolving conductance to the synaptic weights of output neurons, thereby achieving a multi-port input, all-memristive architecture neuromorphic hardware system with spatiotemporal signal processing capabilities.

### SNNs

As a third-generation neural network, SNNs employ spike-based signaling mechanisms that closely emulate BioNN. This biological fidelity manifests through discrete temporal encoding where neurons generate action potentials exclusively when membrane potentials surpass specific thresholds (typically −50 mV to −40 mV). After spike transmission, the membrane potential undergoes rapid repolarization through a rapid change in membrane conductance and returns to the resting state (−70 mV) under the integrate-and-fire mechanism. In Fig. 7G and I, Won et al. [97] constructed an SNN incorporating a LIF neuron implemented through 3-comparator circuitry, featuring 9 memristive synaptic inputs. Through measurements of synaptic weight variations, they experimentally validated the system’s capability for training and classifying binary images, demonstrating that memristive synapses enable STDP via resistance modulation. Experimental results illustrate 2 critical implementations: Temporal encoding mechanisms converting continuous signals into spike trains through adaptive exponential integrate-and-fire models, and hardware realization of LIF [98] neurons using multi-gate memristor circuits. The LIF implementation follows the differential equation: $\tau_{m}\left(\mathrm{dV}/\mathrm{dt}\right)=-\left(V\left(t\right)-V_{\text{rest}}\right)+R_{m}I\left(t\right)$, where $\tau_{m}$ denotes membrane time constant, $V_{\text{rest}}$ is resting potential, and $R_{m}$ represents membrane resistance. As shown in Fig. 7F, Ke et al. [70] constructed an SNN using memristive synapses and threshold-tunable LIF neurons, revealing its ionic dynamics. According to the lithium vacancy conduction mechanism, they used different amplitudes of voltage to excite neurons to realize the adjustment of excitation time, and used the memristor’s ability to retain information under dense time steps to imitate the single-layer SNN’s ability to collect dense pulse signal information. By demonstrating asynchronous sparse bursting, an ultra-low firing rate, and a low discharge rate, they confirmed the high efficiency and low power consumption of SNNs implemented with $Li_{x}\mathrm{Al}O_{y}$ memristors.

Compared to ANNs, SNNs exhibit significantly lower power consumption due to event-driven computation and sparse spike coding. However, their training efficiency remains constrained by temporal dynamics and nondifferentiable spike operations. To address this, Cao et al. [99] developed a novel ANN-to-SNN conversion framework, which involves 2 phases: (a) training standard CNNs with ReLU activation normalization and threshold balancing to optimize weight distributions and (b) replacing ReLU layers with spiking neurons (e.g., LIF neurons) while preserving convolutional kernel weights through synaptic strength mapping. Figure 7E illustrates this conversion paradigm alongside Midya et al.’s [100] implementation using 1T1R crossbar arrays, where nonvolatile memristive devices physically emulate synaptic weight retention and STDP.

## Neuromorphic Learning Implementation via Memristors

### Synaptic plasticity

Synaptic plasticity refers to the activity-dependent modulation of synaptic transmission efficacy, which constitutes the biophysical basis for environmental adaptation and unsupervised learning in neural systems [101]. This phenomenon is governed by calcium ion dynamics and protein phosphorylation cascades in biological synapses. In neuromorphic computing, the learning rules applied to artificial synapses are based on 2 mechanisms: short-term synaptic plasticity (STSP) and long-term synaptic plasticity (LTSP), both of which are inspired by biological neuroscience. In this section, we focus on how memristor devices simulate the learning and memory processes of biological synapses.

**Fig. 8.**
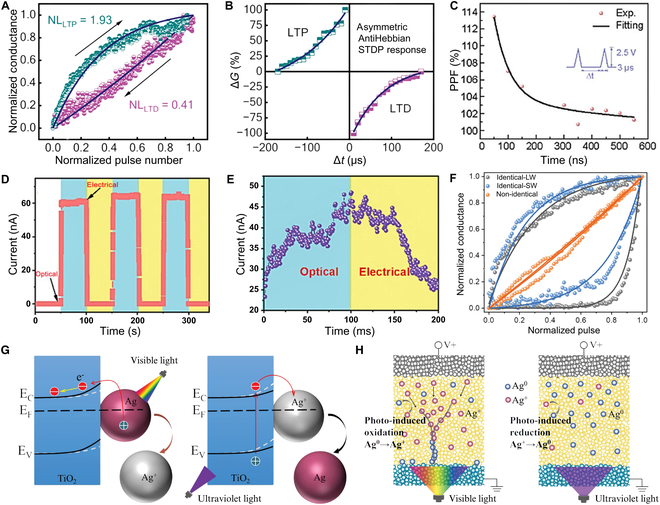
Plasticity of memristor synaptic devices. (A) LTP and LTD characteristics of a $\mathrm{ZnO}/Zn_{2}\mathrm{Sn}O_{4}$ memristor. Reproduced under the terms of the CC BY 4.0 license [104]. Copyright 2023, The Authors, published by Wiley. (B) STDP characteristics of a $\mathrm{ZnO}/Zn_{2}\mathrm{Sn}O_{4}$ memristor. Reproduced under the terms of the CC BY 4.0 license [104]. Copyright 2023, The Authors, published by Wiley. (C) PPF characteristics of an ${\mathrm{HfO}}_{x}$ device. Reproduced with permission from [102]. Copyright 2024, Wiley. (D) Switching characteristics of a carbon-based memristor under optical and electrical signals. Reproduced under the terms of the CC BY 4.0 license [105]. Copyright 2023, The Authors, published by Wiley. (E) LTP characteristics of a carbon-based memristor. Reproduced under the terms of the CC BY 4.0 license [105]. Copyright 2023, The Authors, published by Wiley. (F) LTP and LTD characteristics of a $\text{PdSe}O_{x}/\mathrm{PdS}e_{2}$ memristor. Reproduced under the terms of the CC BY 4.0 license [106]. Copyright 2022, The Authors, published by Wiley. (G) Light-induced synaptic modification of a $\mathrm{Ti}O_{2}/\mathrm{Ag}$ memristor. Reproduced under the terms of the CC BY 4.0 license [140]. Copyright 2021, The Authors, published by Wiley. (H) Electrically driven synaptic modification. Reproduced under the terms of the CC BY 4.0 license [140]. Copyright 2021, The Authors, published by Wiley.

In STSP, the response of a memristor device to single or paired pulses induces an instantaneous but transient change in conductance, thereby simulating the enhancement or inhibition effects of biological synapses over a short time period. As shown in Fig. 8C, Zou et al. [102] simulated biological synapses by fabricating $\mathrm{Hf}O_{x}$ memristors. Under paired pulse stimulation, they measured the device’s paired-pulse facilitation (PPF) index [103], investigating the device’s rapid response to continuous stimuli over a short period. Bridging these findings, the memristor-based STSP characterized by rapid, transient conductance changes demonstrates a promising emulation of biological synapse dynamics.

On the other hand, LTSP involves persistent changes in synaptic weights through repeated or continuous stimulation, providing long-term memory capability for neural networks. It is divided into LTP and LTD. Shrivastava et al. [104] fabricated a $\mathrm{ZnO}/Zn_{2}\mathrm{Sn}O_{4}$ memristor, as shown in Fig. 8A, and verified its LTP and LTD characteristics under electrical stimulation. Figure 8B shows the STDP of the memristor, illustrating that when pulses with different time intervals are applied sequentially, the device’s instantaneous response undergoes rapid changes due to the accumulation of internal ions and charge carriers, leading to persistent weight changes at the synapse. Ai et al. [105] studied a carbon-based memristor, as shown in Fig. 8D and E, which can simulate long-term synaptic plasticity under both optical and electrical signals, achieving data storage for up to 1 month. In Fig. 8F, Li et al. [106] utilized a $\text{PdSe}O_{x}/\mathrm{PdS}e_{2}$-based memristor array to demonstrate the conductance modulation characteristics of LTP and LTD under 3 different pulse schemes, proving that the device can be used for ANN computations. A $\mathrm{Ti}O_{2}/\mathrm{Ag}$ memristor studied by Shan et al. [114] is shown in Fig. 8G and H, where synaptic modifications are achieved via both light-induced and electric-driven mechanisms. This demonstrates that memristors can not only simulate short-term rapid responses but also stably express long-term memory effects under repeated or continuous stimulation, laying the foundation for the application of such memristors in neural network computations for image recognition. Anyway, it is important to note that while STSP reflects the immediate, transient responses of synapses, LTSP captures the enduring modifications essential for long-term memory, together forming a comprehensive framework for neuromorphic learning.

### Learning rules and mechanisms in in-memory computing

**Fig. 9.**
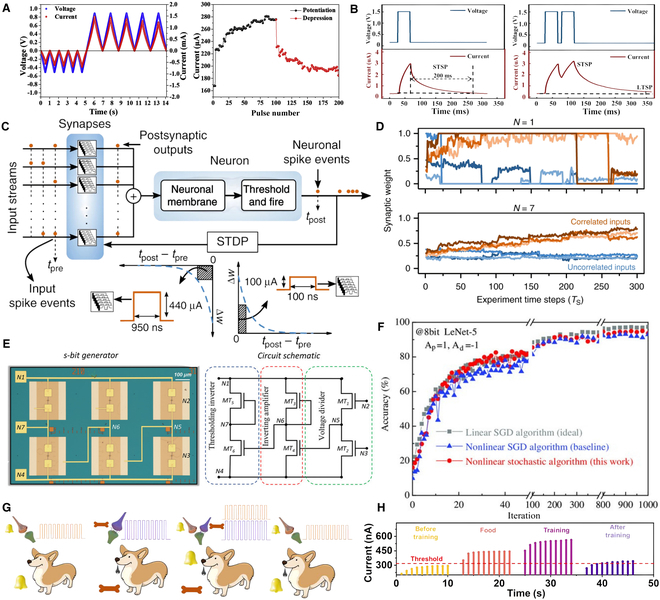
Learning algorithms in in-memory computing architectures. (A) Nonlinear current transport in $Ta_{2}O_{5}$ memristors and LTP/LTD under continuous potentiation/depression spike stimulation. Reproduced under the terms of the CC BY-NC-ND 4.0 license [141]. Copyright 2020, The Authors, published by Elsevier. (B) Plasticity of $\mathrm{Hf}O_{2}/WO_{3}$ memristors under different stimuli. Reproduced with permission from [108]. Copyright 2022, Wiley. (C) Unsupervised learning using multi-phase-change memory architectures. Reproduced under the terms of the CC BY 4.0 license [109]. Copyright 2018, The Authors, published by Springer Nature. (D) Accuracy variation of one synapse implemented with N-phase PCM. Reproduced under the terms of the CC BY 4.0 license [109]. Copyright 2018, The Authors, published by Springer Nature. (E) Optical images and corresponding circuit schematic diagram of the s-bit generator for SC. Reproduced with permission from [114]. Copyright 2022, Wiley. (F) Accuracy of 3 training methods on the MNIST dataset. Reproduced under the terms of the CC BY 4.0 license [79]. Copyright 2021, The Authors, published by Wiley. (G) Pavlovian classical conditioning. Reproduced with permission from [77]. Copyright 2024, American Chemical Society. (H) A memristor array of polyethylene terephthalate/copper ions implementing Pavlovian classical conditioning. Reproduced with permission from [77]. Copyright 2024, American Chemical Society.

Using memristors to construct in-memory computing architectures is an important approach to improving the learning efficiency and energy efficiency of neural networks. In biological synapses, the sliding threshold model reveals the dynamic changes in the sliding threshold induced by LTP and LTD under different firing rates, where an excitation–inhibition balance exists [107]. This phenomenon is crucial for the adaptive adjustment of memristive synaptic weights (Fig. 9A). At the same time, memristors exhibit STSP and LTSP characteristics under different excitation conditions, as shown in Fig. 9B [108], providing a physical foundation for the realization of diverse learning rules.

Unsupervised learning is a type of learning that does not require pre-labeled data, with the goal of enabling neural networks to discover underlying patterns and features in the data without supervision. Memristors exhibit unique advantages in hardware implementations of unsupervised learning, particularly in achieving efficient in-memory computing. As shown in Fig. 9C, Boybat et al. [109] designed a PCM architecture that uses multiple memristors to form synapses. The memory function of the memristors enables weight updates, thereby facilitating unsupervised learning tasks in SNNs. Figure 9D explores the variation of correlated and uncorrelated synaptic weights when different numbers of memristors are used to construct artificial synapses. The study found that multi-device parallel structures offer advantages in unsupervised learning. This provides new insights for designing efficient and stable neural network hardware.

Supervised learning involves training with labeled data, where each input is associated with a fixed output value. The goal is to establish a mapping between the inputs and outputs. Memristors also demonstrate significant application value in supervised learning, particularly in improving training speed and energy efficiency. Zhang et al. [79] designed a hardware-friendly training method based on $1T1R_{1}-1T1R_{2}$ (Fig. 9F), which achieved higher accuracy than traditional stochastic gradient descent (SGD) and nonlinear SGD methods on the MNIST dataset. This adaptive memristor network weight update learning algorithm optimizes the reliance on learning rate parameters in SGD by providing supervision solely on the update direction. With reduced peripheral circuitry requirements and enhanced performance in small-batch training, it realizes a more efficient and energy-efficient CNN training paradigm. By comparing the training accuracy of different algorithms, the effectiveness of memristors in supervised learning within CNNs was evaluated and validated, providing insights for improving training methods in the development of in-memory computing.

Reinforcement learning is a machine learning method in which an agent learns the optimal strategy by interacting with the environment and receiving feedback (rewards or punishments). In memristor-based neural network computing, the application prospects of reinforcement learning are vast. The integrated storage and computation characteristics of memristors make them highly suitable for implementing the policy evaluation and update processes in reinforcement learning algorithms. For example, Wang et al. [110] designed a 3-layer 1T1R memristor array, modifying the learning algorithm for a mixed analog–digital platform and using the RMSprop optimizer to achieve parallel fully connected deep-Q reinforcement learning [111]. Specifically, the 1T1R array is used to perform tasks such as potentiation programming, weight readout, and vector–matrix multiplication.

Associative learning is a type of learning that establishes associations between stimuli and responses, primarily including classical conditioning and operant conditioning. Classical conditioning refers to the process of repeatedly pairing a neutral stimulus with an unconditioned stimulus such that the neutral stimulus eventually triggers the same response as the unconditioned stimulus [112]. Operant conditioning, on the other hand, involves increasing or decreasing the frequency of a behavior through rewards or punishments. A representative form of learning is Pavlovian classical conditioning (Fig. 9G), which, as an important neural behavior in biology, has been widely studied and simulated [113]. As shown in Fig. 9H, Meng et al. [77] used a memristor array made of polyethylene terephthalate/copper ions with multi-channel communication to simulate dendritic synapses. By electrically stimulating the conductive copper ion wires, they replicated the classical reflex experiment of Pavlov’s dog under 2 types of synaptic interactions. This, in turn, enabled high-speed, high-precision ANN-based image recognition.

Stochastic computing (SC) is a hardware-accelerated algorithmic paradigm that replaces traditional binary or floating-point representations with probabilistic signals. Stochastic circuits, which can be interpreted as hybrid analog–digital circuits, leverage stochastic bitstreams for information encoding. This approach mirrors the biological neuronal processing of noisy voltage spike sequences. A defining feature of SC is its capability to perform massively parallel computations at lower precision, making it ideal for energy-constrained neuromorphic systems. Despite the inherent stochastic switching behavior of memristors, which can directly generate random bitstreams, they still rely on CMOS peripherals for accelerated computation. As shown in Fig. 9E, Ravichandran et al. [114] addressed this limitation by developing a memristor-based SC architecture, where they engineered a low-energy, high-precision, and compact-footprint random number generator using the programmable stochasticity of monolayer $\mathrm{Mo}S_{2}$ memtransistors. This work highlights the superiority of non-von Neumann architectures in achieving hardware acceleration. Similarly, Zheng et al. [115] demonstrated a 2D memristive field effect transistor (FET)-based stochastic-bit (s-bit) generator that shares computational kernels with SC, enabling in-memory Bayesian networks for probabilistic reasoning.

## Memristor-Based AI System Applications

Currently, software-based ANNs perform calculations and training using central processing units (CPUs) or graphics processing units (GPUs) based on input data, resulting in high power consumption and low efficiency. Due to their continuously adjustable, nonvolatile conductance modulation characteristics, memristors enable parallel computation and in-memory computing. Therefore, using memristors to simulate the learning, training, and testing processes of ANNs at the hardware level to implement AI system applications has become a popular research direction.

**Fig. 10.**
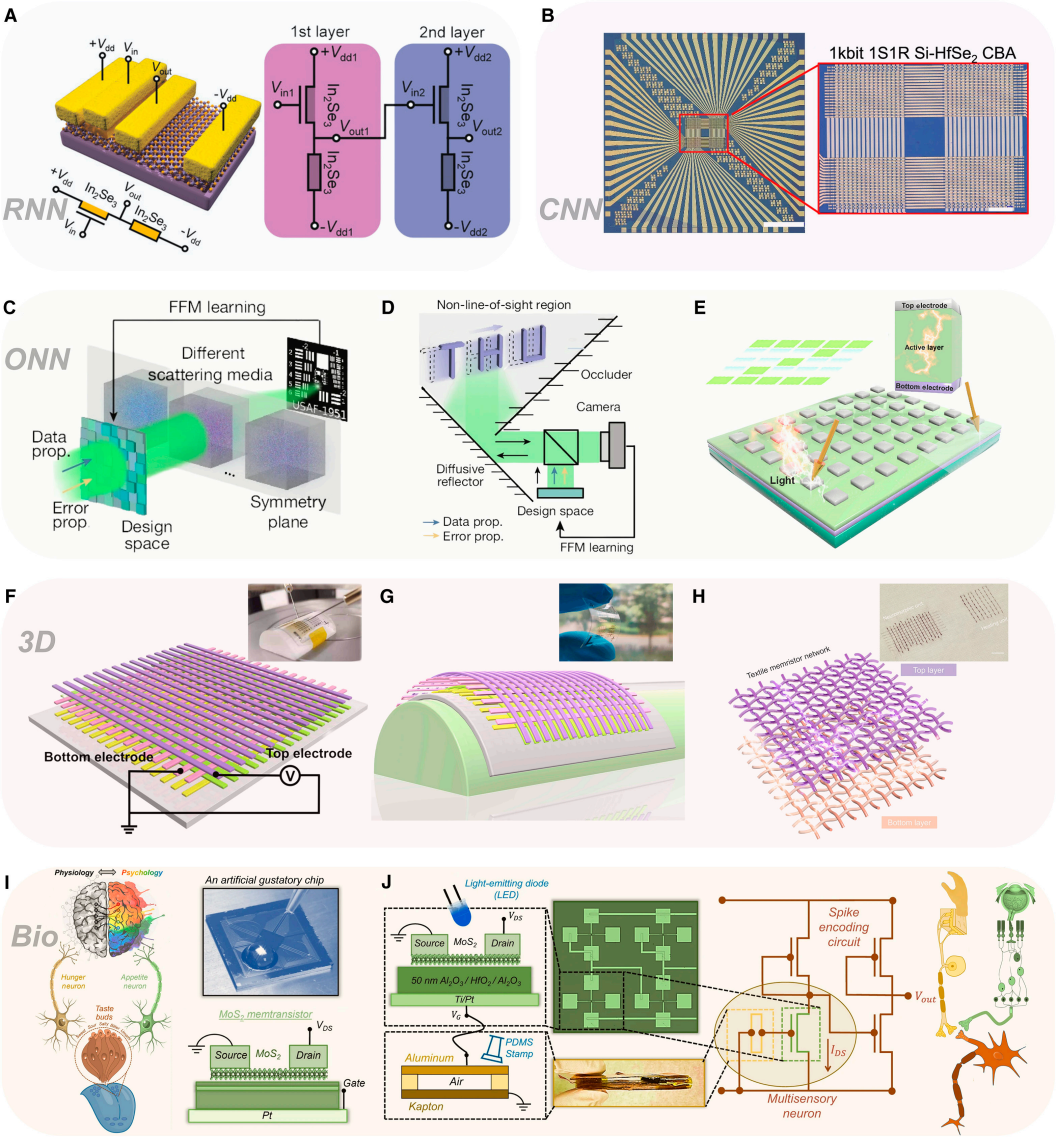
Applications of memristor-based AI systems. (A) A multilayer deep RC architecture based on ferroelectric memristors. Reproduced with permission from [117]. Copyright 2022, Wiley. (B) A 2D memristor crossbar array (CBA) chip enabling fully hardware-implemented binary CNNs. Reproduced under the terms of the CC BY-NC-ND 4.0 license [119]. Copyright 2025, The Authors, published by Springer Nature. (C) The fully feedforward propagation principle of ONNs. Reproduced under the terms of the CC BY 4.0 license [120]. Copyright 2024, The Authors, published by Springer Nature. (D) ONNs for pattern recognition in dynamic scenes. Reproduced under the terms of the CC BY 4.0 license [120]. Copyright 2024, The Authors, published by Springer Nature. (E) An optoelectronic memristor array for sensory computing and pattern recognition. Reproduced with permission from [121]. Copyright 2021, Elsevier. (F) Model and prototype of a flexible synaptic network implemented with a 3D memristor array. Reproduced under the terms of the CC BY 4.0 license [20]. Copyright 2020, The Authors, published by Wiley. (G) Model and prototype of a low-power artificial synapse implemented with a 3D memristor array. Reproduced with permission from [82]. Copyright 2020, American Chemical Society. (H) Model and prototype of a reconfigurable textile neural network implemented with a 3D memristor array. Reproduced under the terms of the CC BY 4.0 license [44]. Copyright 2022, The Authors, published by Springer Nature. (I) A 2D bio-inspired gustatory circuit implemented with monolayer $\mathrm{Mo}S_{2}$ memtransistors. Reproduced under the terms of the CC BY 4.0 license [123]. Copyright 2023, The Authors, published by Springer Nature. (J) A bio-inspired visuotactile neuron based on a photosensitive monolayer $\mathrm{Mo}S_{2}$ memtransistor and a triboelectric tactile sensor. Reproduced under the terms of the CC BY 4.0 license [124]. Copyright 2023, The Authors, published by Springer Nature.

In the field of RNN computing, RC has attracted considerable attention due to its low training cost, since only the readout network requires training, thereby spurring interest in the hardware implementation of RC networks using memristors. Midya et al. [67], capitalizing on the fixed initialization of weights between the hidden layer and the reservoir, focused on training the weights from the reservoir to the output layer and proposed a row-wise programming 1T1R array based on $\mathrm{Si}O_{x}/\mathrm{Ag}$ memristors. Owing to the high nonlinearity of this in-memory computing unit, system training becomes more efficient and finds extensive applications in temporal pattern classification. Chen et al. [116] exploited the excellent controllability of ferroelectric memristors by constructing the reservoir with volatile ferroelectric memristors and the readout network with nonvolatile ferroelectric memristors, thereby realizing an all-ferroelectric RC system capable of curvature discrimination and digit recognition. In Fig. 10A, Liu et al. [117] connected a ferroelectric semiconductor field-effect transistor in series with a ferroelectric resistor to implement a single-layer RC circuit. By leveraging the dynamic voltage divider effect and cascading capability, they constructed a multi-layer reservoir system. This approach not only enhanced low-pass filtering, short-term memory capacity, and hierarchical information processing but also represented a significant breakthrough in utilizing 2D materials for multi-layer reservoir components. These findings pave the way for the development of deep RNN architectures.

In the realm of CNN computing, Yao et al. [118] proposed an ultra-low-power, purely hardware-based structure utilizing a 1T1R array to achieve in-memory computing. This design optimizes computation speed and training cost while reducing convolutional delays through the parallelization of multiple convolution kernels, offering new perspectives for future memristor-based neural network computing. Building on this, in Fig. 10B, Jain et al. [119] implemented a fully hardware-implemented binary CNN using 1S1R arrays, mitigating challenges associated with 2D memristor arrays such as limited scalability and high sneak currents. As a stepping stone for weight training in SNNs, schemes that transfer weights trained on ANNs to SNNs have been widely explored. For instance, Cao et al. [99] replaced the ReLU neurons in CNNs with spiking neurons, configuring synaptic strengths to mimic convolutional kernel weights; likewise, Midya et al. [100] designed a CNN compliant with SNN architecture by employing a 1T1R array to implement an activation function HalfRect conversion structure. Both approaches offer novel insights for the evolution of third-generation neural networks.

In the field of optical neural network (ONN) computing, Xue et al. [120] proposed a fully feedforward mode (FFM) that implements the training process entirely in hardware. As shown in Fig. 10C, the introduction of gradient-based FFM enhances both optimization efficiency and imaging quality; Fig. 10D further demonstrates that ONNs employing FFM can perform imaging and classification tasks on dynamic scenes, thereby providing theoretical support for more efficient hardware training paradigms. Although ONNs can use optical modulators, wave conductors, photodetectors to realize photonic operations, and wave division multiplexers to merge or separate signals, it is still a promising research direction to use memristors (including RRAM and PCM) combined with ONN basic structure to realize ONN computing with real-time sensing and nonvolatile storage characteristics. The photonic memristor array (Fig. 10E) proposed by Wang et al. [121] for sensing computing and pattern recognition has in situ optical sensing and storage capabilities, which promotes the development of ONN in sensor-based artificial vision systems.

In the domain of 3D wearable neural network computing, the research team led by Chen [20] has made significant contributions. Their work proposed a 3D memristor array structure based on ternary oxides (Fig. 10F), wherein the memristor serves as the smallest storage unit to enable multi-bit storage. Such a high-performance storage architecture provides a reliable hardware platform for large-scale neural network computing. Furthermore, owing to its flexible and foldable properties, this memristor array is well suited for application in wearable neuromorphic devices. The team has also contributed to the implementation of synaptic functionalities in crossbar memristor arrays (Fig. 10G), achieving ultra-low-power synaptic plasticity that mimics the biomimetic characteristics of 3D ANN systems under multi-synaptic regulation [82]. Additionally, the team introduced a textile memristor network with a reconfigurable simplified neuron structure (Fig. 10H), which achieves system-level integration of a thermal control module, thereby promoting the advancement of memristors in the field of intelligent IoT [44]. Oh et al. [61] fabricated a polyvinyl alcohol-based memristor capable of spike-dependent learning processes, advancing the application of organic memristors in constructing flexible synaptic components for wearable intelligent systems. Son et al. [122] integrated oxide-based RRAM arrays with nanoscale metal nanoparticle switches into a nanomembrane platform, serving as a memory array for highly stretchable wearable bio-integrated systems. This work pioneers system-level biomedical applications of memristors by enabling closed-loop diagnosis–therapy integration, offering a critical solution for real-time biosignal monitoring and responsive treatment actuation.

In the field of bio-inspired neuromorphic computing, Ghosh et al. [123] developed an artificial gustatory chip by modeling the taste cortex with a monolayer $\mathrm{Mo}S_{2}$ memtransistor and the tongue with a graphene chemitransistor. As illustrated in Fig. 10I, when the tongue samples a food, its various components elicit distinct stimulus signals at taste receptors. These signals are conveyed synaptically to “hunger neurons” and “appetite neurons”, which are then processed by the brain and sent back to the receptor cells on the tongue to elicit the appropriate feedback. Within this biomimetic gustatory system, memtransistor-based comparators exploit nonvolatility and programmable conductance to adjust the brain’s hunger and satiety thresholds. Basic logic gates built from memtransistors then realize a bio-inspired, adaptive feeding circuit that integrates analog and digital computation, effectively merging taste–cortex and brain–cortex functions. Moreover, Sadaf et al. [124] proposed an artificial visuo-tactile system based on a photosensitive memtransistor, enabling multimodal sensory integration akin to the brain. In Fig. 10J, they connect a tactile sensor to the gate of a photosensitive memtransistor to produce both tactile pulses and photo-electric threshold shifts. This dual control modulates the drain–source current, which is then encoded into digital spike trains, realizing a multimodal output driven by both touch and vision. Biomimetic neuromorphic systems have made breakthroughs in emulating multisensory perception and adaptive behaviors. Beyond human-inspired designs, researchers have drawn from animal models: Zheng et al. [125] built a multisensory platform integrating visual and chemical cues inspired by butterflies; Das et al. [126] leveraged avian interaural time differences in low-frequency sound for spatial hearing using 2D transistors; and Jayachandran et al. [127] emulated the locust’s lobula giant movement detector neuron to create a low-cost, low-power collision sensor. Advances in bio-inspired accelerators are also elevating the energy efficiency of neuromorphic hardware. Subbulakshmi Radhakrishnan et al. [128] constructed a digital encoder using a photonic memtransistor to accelerate SNN hardware based on spike timing and sparse optical coding, demonstrating the method’s feasibility for the first time. Sebastian et al. [129] addressed the limited programmable conductance states in memristive simulated annealing by integrating memtransistors with a 1T1R architecture, realizing hardware-accelerated annealing for lsing spin glass systems.

## Challenges and Outlook

Memristor-based neural network computing technology faces multiple challenges, among which energy efficiency and power consumption are particularly prominent. Although many neuromorphic computing devices have improved energy efficiency compared to traditional memory, they still cannot match the ultra-low power consumption of biological synapses. For example, the research conducted by the Wang’s team [130] provides new insights to address this challenge. The heterogeneous synapse, based on flexible MoS2 memristors designed by their team, enables a simplified neural network structure, achieving a per-spike energy consumption of less than 30 aJ under optoelectronic co-regulation. The flexible memristor synapse based on boron nitride designed by the Meng’s team achieves a per-spike energy consumption of less than 200 fJ, with the additional advantage of a response speed of 1 μs [131]. This lays the foundation for low-power, high-efficiency, and durable wearable memristor-based neural network computing. In terms of materials and fabrication processes, although there has been research on memristor textiles, challenges remain in designing advanced memristive materials and achieving high-quality active layers on fiber electrodes, making efficient computation difficult to achieve. The memristor based on DNA bridging studied by Xu’s team, using electrophoretic deposition technology to form a uniform film of DNA molecules on metal fibers, combined with silver nanoparticles, achieves ultra-low operating voltage, high-speed switching, and low power consumption [132]. This allows the memristor to outperform traditional organic memristors in terms of performance, opening up new avenues for the development of textile memristor materials. Circuit design and system integration are also important challenges. Currently, ANN systems based on the von Neumann architecture require higher system power consumption and area due to the separation of computation and memory units.

The integration of memristor-based in-memory computing neural networks with CMOS technology remains a challenge. Many high-performance memristive materials, including perovskite oxides and certain 2D layers, require high-temperature processing that is incompatible with standard CMOS back-end manufacturing and therefore cannot be directly integrated [58]. To address this issue, one can either improve the memristor fabrication process or develop new memristive devices using materials that are compatible with CMOS technology. The memristor process based on SiCO:H, studied by the He team, is compatible with CMOS [133] and is expected to replace the analog-to-digital conversion module, enabling the integration of memristors on a chip. Memristors must also exhibit uniform device variability, low forming voltages, minimal cycle-to-cycle variation, and robust thermal and electrical stability to satisfy CMOS reliability requirements. The absence of standardized process design kits and accurate electronic design automation models further complicates circuit development. Even more critically, uncontrolled defect distributions in amorphous or polycrystalline switching layers cause inherent stochastic switching behavior, resulting in significant device-to-device and cycle-to-cycle inconsistencies. Even minor performance fluctuations in each device over time impede precise programming and reliable state retention across the entire array, rendering large-scale training impractical. Finally, achieving very fast switching speeds and low-voltage operation is essential for low-power CMOS integration. Addressing these thermal, variability, and tooling challenges will be pivotal for realizing scalable, high-density, and commercially viable memristor–CMOS hybrid neuromorphic systems.

**Fig. 11.**
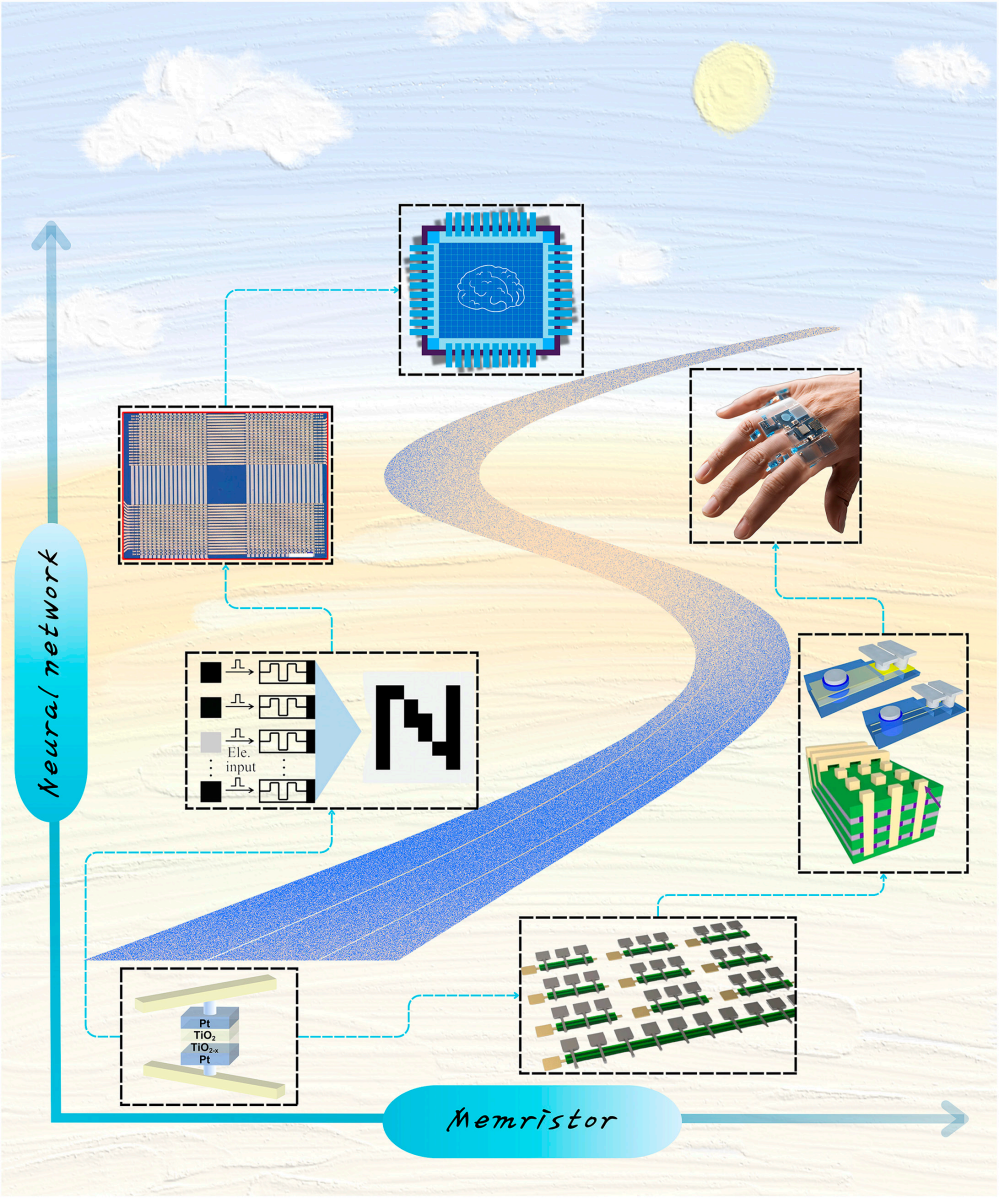
Conceptual diagram of the future directions in the development of memristors and neural networks. The conceptual development of memristors, along with their experimental validation, emerged relatively later compared to that of neural networks. However, once discovered, memristors were rapidly integrated with mature CMOS technology, enabling the fabrication of 2D crossbar arrays at sub-nanometer scales (reproduced under the terms of the CC BY 4.0 license [21]; copyright 2022, The Authors, published by Springer Nature), and subsequently, 2-layer 3D RRAM (reproduced under the terms of the CC BY 4.0 license [142]; copyright 2015, The Authors, published by Springer Nature) and 3D vertical crossbar array memristors (reproduced under the terms of the CC BY 4.0 license [18]; copyright 2016, The Authors, published by Springer Nature). This advancement provides the physical basis and methodology for enhancing efficiency and achieving ultra-dense integration in neural network hardware, with promising prospects for wearable devices and edge computing in the future. Currently, the integration of functions such as visual sensing and pattern recognition into memristor-based hardware implementations of CNNs and SNNs (reproduced under the terms of the CC BY 4.0 license [140]; copyright 2021, The Authors, published by Wiley), along with the design of fully hardware-implemented 1S1R-based CNN chips (reproduced under the terms of the CC BY-NC-ND 4.0 license [119]; copyright 2025, The Authors, published by Springer Nature), lays the foundation for countering machine learning attacks to maintain hardware security and for the chip-level application of brain-inspired neural computing.

Memristor-based neural network computing has a promising future. Figure 11 gives an overview of the future development directions of memristor and neural networks at the application level: flexible neuromorphic devices and brain-inspired neurocomputing chips. First, technological breakthroughs and performance improvements are key directions for future development. With the continuous optimization of fabrication processes, key parameters such as the stability, linearity, switching ratio, and durability of memristors are expected to improve significantly, which will greatly enhance the performance of memristors. Currently, issues such as process fluctuations and noise interference still exist in in-memory computing chips, and future breakthroughs in this area will undoubtedly be one of the driving forces for development. Research on flexible memristor arrays, especially their application in simulating synaptic plasticity and high-density storage, has demonstrated their potential in neuromorphic computing for wearable devices. In terms of memristor material and structure development, such as altering the charge capture layer thickness and the 1TNR structure, these are avenues for expanding ideas that are worth exploring in future research. Algorithm development and application expansion will be another important direction for future research. Currently, SNNs represent an architecture closer to BioNNs, offering superior advantages in emulating neurobiological systems. Memristors, leveraging their memristive properties, demonstrate exceptional potential in in-memory computing architectures. Although memristor-based hardware implementations of SNNs remain limited, emerging studies have begun to reveal their unique benefits. Future research will focus on spiking neurons and SNN algorithms that can be implemented in hardware, which will not only enhance the performance of neural networks in dynamic and complex tasks but also enable memristors to be widely used in more practical applications.

Meanwhile, industrial deployment of memristor-based neural network hardware is accelerating. Knowm Inc. now offers a commercial “memristor discovery” board for neuro-memristive prototyping, enabling rapid evaluation of memristive synapse and neuron circuits. Crossbar Inc. provides an RRAM-based AI accelerator chip that integrates dense passive memristor arrays with programmable comparator logic over a serial peripheral interface interface, optimized for content-addressable matching operations at the end of deep neural network inference. In China, laboratory-stage developments are even more remarkable: Tsinghua University’s realization of the world’s first fully system-integrated memristor-based CNN in-memory computing chip undoubtedly demonstrates the feasibility of large-scale memristive crossbar integration for high-performance, energy-efficient on-chip neural network inference and learning [118]. Beyond memristive devices, photonic AI accelerators are entering production: The Taichi photonic chiplet demonstrates 160 TOPS/W energy efficiency in a diffractive-interference architecture, and integrated ONNs achieve sub-nanosecond latencies and terahertz-scale parallelism in compact silicon photonic platforms [134]. Xizhi Tech’s PACE photonic co-processor, featuring an optical matrix multiplier integrated with CMOS electronics in a 3D-stacked package, performs sub-nanosecond multiply-accumulate operations at 1 GHz and accelerates deep convolutional inference by up to several hundred times compared to high-end GPUs, demonstrating the commercial viability of ONN accelerators [135]. Prototypes such as Fathom Computing’s optical AI trainer leverage pulsed laser-based matrix multiplications to train neural networks entirely in photonic hardware, achieving real-time handwritten digit recognition with high accuracy while consuming orders of magnitude less energy per operation than GPU-based systems. These results demonstrate the commercial potential of optical computing for scalable, energy-efficient AI training platforms. Finally, the integration of silicon photonics and nonvolatile memristors offers an attractive solution for overcoming the von Neumann bottleneck [121,136]. Photonic memristors, characterized by their energy efficiency, nonvolatility, and scalability, have rapidly advanced in fields such as optical neuromorphic computing, brain-inspired photonic networks, and optical computing architectures.

## Data Availability

The data that support the findings of this study are available from the corresponding author upon reasonable request.
